# Intestinal Barrier Interactions with Specialized CD8 T Cells

**DOI:** 10.3389/fimmu.2017.01281

**Published:** 2017-10-11

**Authors:** Špela Konjar, Cristina Ferreira, Birte Blankenhaus, Marc Veldhoen

**Affiliations:** ^1^Instituto de Medicina Molecular, Faculdade de Medicina da Universidade de Lisboa, Lisbon, Portugal

**Keywords:** mucosal immunology, intraepithelial lymphocytes, inflammatory bowel disease, CD8^+^ T-lymphocytes, epithelial cells

## Abstract

The trillions of microorganisms that reside in the gastrointestinal tract, essential for nutrient absorption, are kept under control by a single cell barrier and large amounts of immune cells. Intestinal epithelial cells (IECs) are critical in establishing an environment supporting microbial colonization and immunological tolerance. A large population of CD8^+^ T cells is in direct and constant contact with the IECs and the intraepithelial lymphocytes (IELs). Due to their location, at the interphase of the intestinal lumen and external environment and the host tissues, they seem ideally positioned to balance immune tolerance and protection to preserve the fragile intestinal barrier from invasion as well as immunopathology. IELs are a heterogeneous population, with a large innate-like contribution of unknown specificity, intercalated with antigen-specific tissue-resident memory T cells. In this review, we provide a comprehensive overview of IEL physiology and how they interact with the IECs and contribute to immune surveillance to preserve intestinal homeostasis and host-microbial relationships.

## Introduction

The intestinal epithelia are a single cell layer of large surface. Together with a mucus layer, the epithelia form a dynamic physical barrier between the host and its environment. Estimates are up to 100 trillion microorganisms, including pathogens, have made the gastrointestinal tract their home (1), which makes the intestine the largest potential port for microbial invasion. However, a proportion of the microorganisms in the intestine can contribute to the hosts’ health and immunity. These commensal bacteria compete for resources with pathogenic microorganisms and provide metabolic capacity to digest food products by generating important compounds (e.g., vitamin K) or by assisting other microorganisms with supportive roles. The delicate nature of the single cell epithelial barrier, the essential function of the gastrointestinal tract to absorb nutrients and liquids, and the balance to maintain beneficial microbes, while offering protection against invasion and avoiding tissue damage, requires an effective and robust, yet tolerant, immune system.

The intestinal immune surveillance network is an integrated part of the organ, which enables it to swiftly pick up cues regarding its health status and contributes to tissue homeostasis as well as repair. Immune surveillance links rapid activation of innate immune cells to the more delayed recruitment of adaptive immune cells (2), ultimately resulting in immunological memory. Part of the innate system is the intestinal epithelial cells (IECs) themselves as well as classical innate immune cells. Mostly, macrophages, monocytes, and dendritic cells (DCs) migrate to the intestine from the bone marrow *via* blood (3). Following infection, interactions between antigen presenting cells and lymphocytes can take place in specialized structures, unique to the intestine, such as isolated lymphoid follicles and Peyer’s patches (4).

T-lymphocytes recognize foreign particles (antigens) by their surface expressed T cell receptor (TCR). With each T cell expressing a nearly unique TCR, collectively T cells can recognize nearly all foreign antigens. From the two major types of T cells found in blood and secondary lymphoid organs (SLO), CD4 expressing helper T (T_H_) cells are generated in the thymus as precursors without a defined function. They recognize antigens presented in major histocompatibility complexes class II (MHCII) after processing by antigen presenting cells. T_H_ cells have an important orchestrating role, differentiating into effector cells with distinct supportive functions in type 1 (T_H_1), type 2 (T_H_2), and type 3 (T_H_17) immunity and high levels of flexibility (5, 6). Specialized regulatory T cells can curtail responses and form part of a carefully balanced immune system (7). CD8 expressing cytotoxic T cells similarly derive from the thymus as naive cells. They mainly recognize antigens resulting from the target cells’ transcriptional machinery and degradation of cytosolic proteins by the proteasome presented in MHCI, such as those resulting from viral infections as well as intracellular bacterial infections. Upon encountering their cognate antigen, CD8^+^ T cells differentiate into effector cells, classically thought to be part of type 1 immunity due to their high potential for interferon (IFN)γ production.

**Figure 1:**
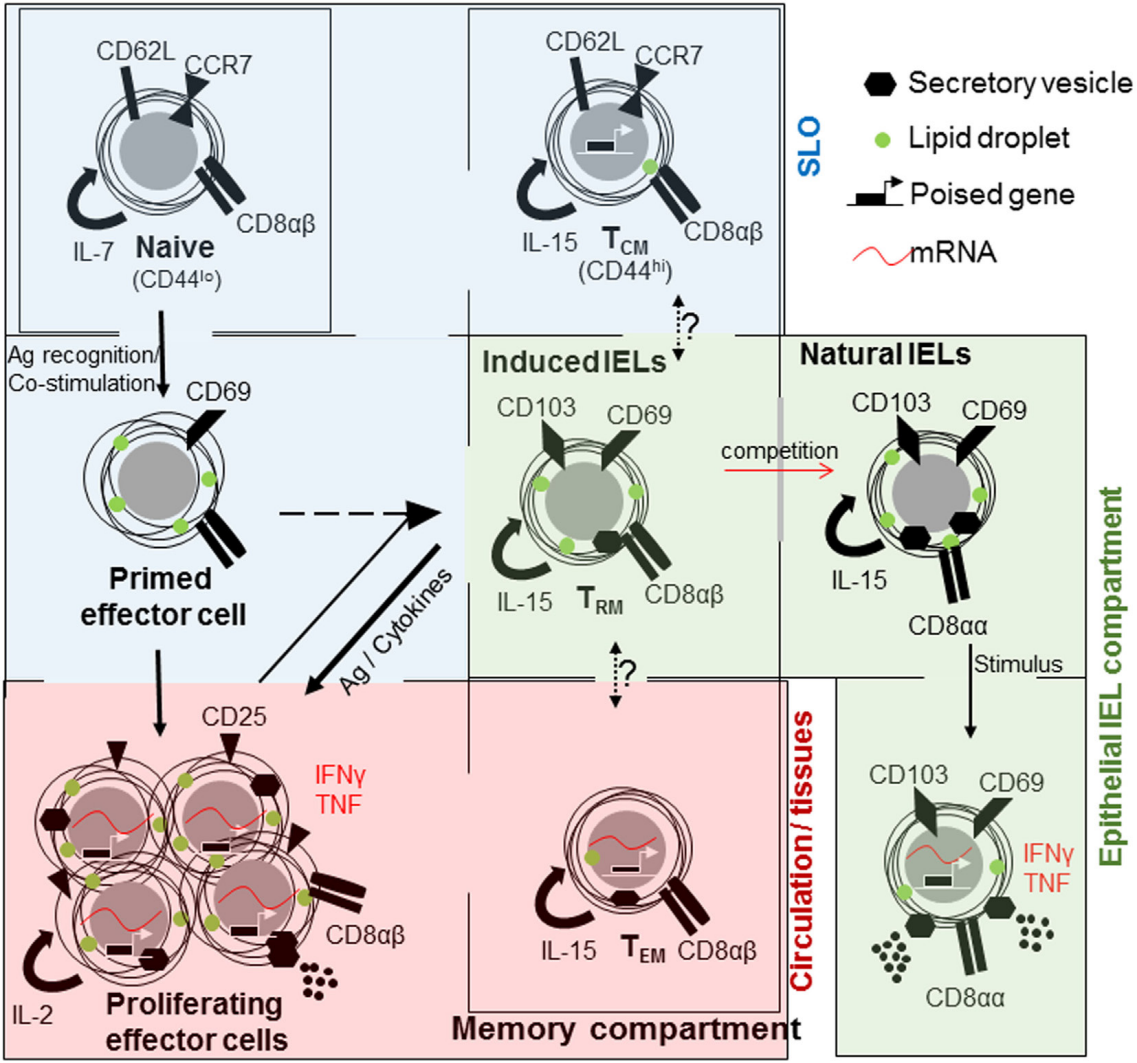
The relationships between CD8^+^ T cell populations in the small intestine. Naive CD8^+^ T cells (top left) are maintained in a quiescent state within their own compartment under homeostatic control. They mainly circulate through the secondary lymphoid organs (SLO). Upon encountering antigen, T cells are primed, acquire cellular building blocks such as lipids, and express CD69. Thereafter, they undergo rapid proliferation and express CD25 [high affinity interleukin (IL)-2 receptor], cytokines such as tumor necrosis factor (TNF) and interferon (IFN)γ and can release cytolytic factors, as effector T cells. Large proportions or effector T cells will die by apoptosis. Memory cells are derived from primed or effector T cells of which three subsets are distinguished; central memory T cell (T_CM_) that is present in the SLO, effector memory T cells (T_EM_) that are circulating and rapidly acquire effector functions and tissue-resident cells (T_RM_) in tissues, especially barrier sites, such as the skin and intestine. All memory cells rely on IL-15 for their maintenance. At barrier sites T_RM_ cells compete with natural intraepithelial lymphocytes (IELs), both maintained in a semi-activated state expressing CD69 and CD103 and metabolically charged.

The maintenance of effector T cells is metabolically costly. Rapidly dividing cells require large amounts of energy for the production of cellular building blocks and secretion of effector molecules. These cells can potentially contribute to chronic inflammation and immunopathology. To avoid such possible danger and energy expense, the majority of effector cells undergo apoptosis after pathogen clearance, re-establishing homeostasis. Yet, some persist as memory cells, providing protection against re-infection. Memory CD8 T cells are a heterogeneous population, varying in phenotype, function, and localization (8) (Figure 1). This facilitates a swift and tailored response to a broad array of potential insults. In addition, the intestinal immune system has another important population of specialized CD8^+^ T-lymphocytes known as intraepithelial lymphocytes (IELs) (9). Intriguingly, IELs have characteristics of naive, effector, and memory cells require bidirectional cross-talk with IECs (10) (Figure 1), with one murine IEL estimated to be present for every 4–10 IECs (11, 12).

Aberrant immunity has severe consequences, especially in the intestine where a single epithelial cell layer forms the barrier between the host and a very high amount of microorganisms. Immunity against commensal bacteria can result in chronic inflammation, such as observed in inflammatory bowel diseases (IBDs). In this review, we focus on CD8 expressing T cells, particularly IELs, which, located in the very top layer of the intestinal barrier, are ideally positioned to monitor the intestinal microbiota. They may contribute to modulating immunity toward microbes as well as immunopathology, and are involved in tissue homeostasis and epithelial repair. We will discuss some of the properties of IELs and speculate on their role in the intestinal immune surveillance network.

### Conventional CD8 T Cells

The initiation of an adaptive immune response requires several myeloid and lymphoid cell types. These cells need to be brought together and act in a strictly orchestrated manner in time and space to license immune cell activation (13). Critical interactions are those between antigen presenting cells, especially DCs and T cells (14). In order to become fully activated, naive T cells require signaling through TCR (signal 1) as well as costimulatory receptors (signal 2), such as CD28 and CD40. Additional cues (signal 3) provide inflammatory context and involve cytokines and chemokines (15, 16).

During the initiation phase, naive CD8^+^ T cells rapidly proliferate and differentiate into cytotoxic T-lymphocyte effector cells thereby gaining the ability to kill target cells by releasing perforin and granzymes, and secrete large amounts of cytokines, such as tumor necrosis factor (TNF) and IFNs (17) (Figure 1). The rapid proliferation during the expansion phase ensures that a limited number of precursor cells can counter infectious agents. Effector cells migrate to most tissues in the body to ensure the removal of all infected cells and pathogens (18). However, such a response cannot be sustained and proximally 95% of effector cells die in a contraction phase upon pathogen clearance (19).

A limited number of cells develop into memory cells, returning to a state of quiescence with slow cell turn over and effector molecule transcription. Despite this they are able to rapidly reactivate, proliferate, and express effector molecules upon reencounter with a similar pathogen (18, 20–23). How memory T cells develop remains incompletely understood. There are different signals influencing T cells upon and after encountering their cognate antigen that influence the size and quality of the T cell memory pool (8).

Three subtypes of memory T cells are recognized, they are; effector memory (T_EM_) T cells, central memory (T_CM_) T cells (18, 24, 25), and tissue-resident memory (T_RM_) cells expressing CD69 and CD103 (26–29) (Figure 1). Differences in cell localization, recall ability, and effector functions provide intersecting levels of protection against re-infection (30). Memory cells found circulating through blood, lymph, and SLO are referred to as T_CM_ cells and express CD62L and CCR7, which enable entry in lymphoid organs and circulation (31–34). Those cells primarily found in non-lymphoid tissues are T_EM_ cells (18, 22).

Although migration of T cells is a pillar of successful immune defense, experiments using defined tissue grafts from ganglia, skin, and intestine as well as the use of parabiosis have defined a residential population of memory T cells (27, 35–37). At epithelial barrier sites such as the skin, lungs, reproductive organs, and gastrointestinal tract, a unique memory population is found; T_RM_ cells. These cells share characteristics with T_EM_ cells, expressing CD44 and low levels of CD62L (Figure 1). They are found at the initial site of infection, providing very regional immune surveillance and protection against re-infection (35, 38, 39), and do not recirculate (40). The discovery of T_RM_ cells and subsequent detailed analysis have resulted in a paradigm shift that most memory T cells are an integral part of non-lymphoid tissues (41). But, these cells did not settle on empty ground to fill a previously non-existent niche. T_RM_ cells compete, successfully, with innate or innate-like lymphocytes, which are already present at the original site of infection (39).

### Intraepithelial Lymphocytes

A variety of innate or innate-like lymphoid cell types reside in tissues, including natural killer (NK) cells, innate lymphoid cells, and T cells expressing the γδ TCR chains (γδ T cells), homodimers of CD8α or a semi-invariant TCRαβ such as NKT cells, and mucosal associated invariant T cells. The top layer of the epithelia, in murine and human intestine as well as murine skin, contains large populations of such innate-like T cells within the IEL population.

Intestinal IELs express the prototypical tissue-resident integrin CD103 (integrin αE), with which they interact with IECs (10), as well as C-Type lectin and early activation marker CD69, and the NK cell inhibitory receptor 2B4 (CD244) (42). Antibody staining for CD8α, CD69, and CD103 in lymphocytes sourced from the intestinal intraepithelial fraction provides a homogenous cell population (42). However, IELs can be divided into subsets based on their activation mechanism and on the antigens, which they may recognize. Induced or adaptive IELs are derived from conventional CD8αβ T cells, which recognize non-self antigens in the context of MHCI. They home to the intestinal barrier upon encountering their cognate antigen in the intestine as T_RM_ cells (9, 43). Induced IELs accumulate with age (44), replacing natural IELs (39).

Natural or innate-like IELs also originate in the thymus where they acquire homing factors and identity upon selection on self-antigens and seed the intestine as a precursor population (45–51). They express CD8αα homodimers, in contrast to conventional CD8αβ T cells (52) (Figure 1). They express either the conventional αβ TCR or the non-conventional γδ TCR. In the small intestine around 60% of all IELs express TCRγδ, in marked contrast to SLO in which γδ T cell represent less than 1% (53). In humans, natural IELs predominantly express TCRVδ1 with a contribution from TCRVδ3, the majority of which express CD8 (54, 55). In contrast to murine cells, human γδ T cells may process and present antigen (56).

Contrary to T cells found in SLO and in line with T_RM_ cells, IELs do not circulate through blood and lymph and are tissue-resident (57). IELs seem to respond to a broad range of inflammatory cues, but the precise identity of these signals remains unknown. They modulate epithelial cell homeostasis and local immune responses by targeting other immune cells, viruses, and bacteria (9, 58, 59). The majority of IELs hold cytoplasmic granules containing large amounts of granzymes, cytokines, and chemotactic factors (42, 43, 60–62). At the first sight, the cytotoxic properties of IELs suggest, they can cause damage to the epithelial barrier by powerfully attacking infected cells, particularly IECs (63). However, IELs are well adapted to the intestinal environment in order to survive and perform their functions in protecting the delicate epithelial layer. In recent years, several studies of IELs have revealed distinct characteristics regarding their maintenance, activation, and contribution to the host immune response to preserve a healthy epithelial barrier.

### Maintaining IELs

Intraepithelial lymphocytes develop pre-birth, occupy the epithelia before microbial colonization, and play an important role in immune protection during early life (64). It remained debatable for a considerable time if natural IELs take up residence at the intestinal epithelia as precursor naive-like cells or as antigen-experienced memory-like CD8^+^ T cells poised for activation or reactivation. The later would suggest that a priming step may be required, post-thymic development in the SLO, before seeding in IEL compartment. Transcriptional analysis, comparing IELs harvested under non-inflammatory conditions with memory CD8^+^ T cells, revealed paradoxical findings of their activation status (42, 43). IELs constitutively express transcripts of genes associated with activated cytotoxic T cells [granzyme A, granzyme B, serglycin, Fas ligand (FasL), and CCL5]. Yet, at the same time, IELs highly express transcripts of genes involved in immune regulation. These include cytotoxic T-lymphocyte associated protein 4, Ly49E-G, the NK cell inhibitory receptor Ig superfamily-related gp49B, and programmed cell death 1 (42). Factors involved in microbe-toxicity, such as regenerating islet-derived protein 3 gamma (Reg3γ), are readily detected in IELs under steady state conditions (65). In addition, several transcripts have been translated, and proteins are present and stored in secretory vesicles, e.g., granzymes (66). IELs home are retained in a poised activation state in mice lacking most secondary organs (67, 68), suggesting priming in secondary organs for natural IELs is not essential and the IEL activation status may be maintained by factors in the local epithelial environment.

Natural IELs are present in axenic mice. However, reduced numbers (of induced IELs) and decreased cytotoxicity of IELs from germ-free mice indicate that signals from the microbiota or other environmental stimuli are required to maintain intestinal CD8^+^ T cells and their function (51, 69, 70). The ligand activated transcription factor, arylhydrocarbon receptor is critical for IEL maintenance (59, 71). Of interest, the provision of ligands can be achieved *via* food intake, especially green vegetables, and may also be obtained from the microbiota (59, 72). Curiously, 30–50% of IELs from conventional standard pathogen-free mice express the marker Thy1 (CD90), but those found in axenic mice do not. Colonization of germ-free mice results in the generation of Thy1-expressing IELs (69), but as yet no functional differences have been attributed to the expression of Thy1.

**Figure 2:**
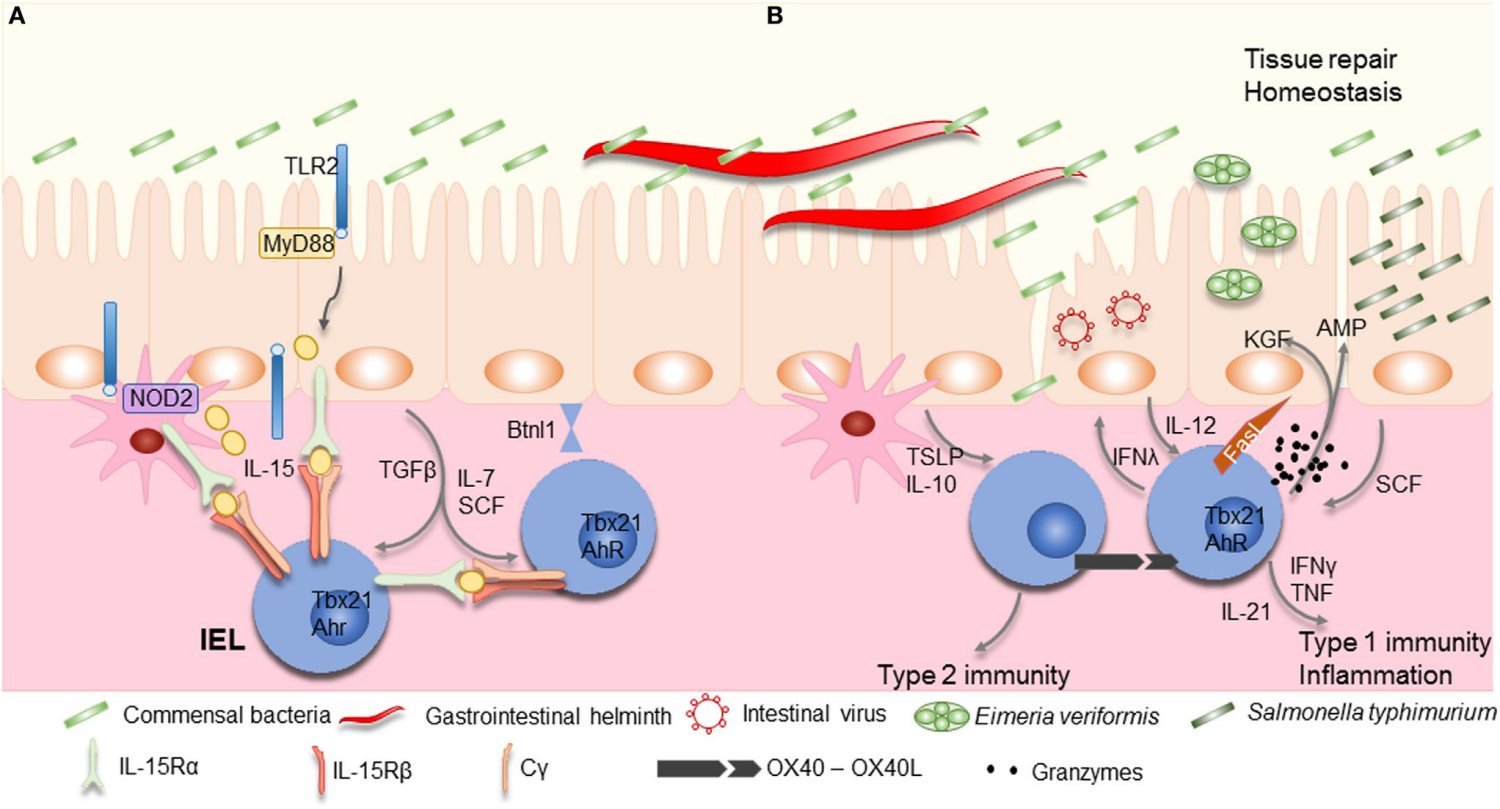
Maintenance and activation of intraepithelial lymphocytes (IELs). **(A)** Commensal bacteria can contribute to IEL maintenance. Signaling *via* TLR2 and myeloid differentiation primary response gene 88 (MyD88) increases interleukin (IL)-15 production, an important survival factor for IELs. Antigen presenting cells, such as dendritic cells (DCs) or macrophages, also produce IL-15 in a NOD2 dependent manner. IL-15 is bound to the IL-15Rα on the producing cells, and is presented *in trans* to the IEL, which carry the IL-15Rβ/Cγ chain receptor complex, and signals *via* the transcription factor Tbx21. IL-7 and stem cell factor (SCF) are additional examples for IEC derived cytokines important for IEL survival, while arylhydrocarbon receptor expression (AhR) and tissue-specific factors, such as butyrophilin-like 1 (Btnl1), play an additional role in maintaining IELs. **(B)** Infections cause disruption or damage to the epithelial barrier. Dependent on the type of insult, IEC and DCs produce cytokines like thymic stromal lymphopoietin (TSLP), IL-10, IL-12, or SCF, thereby directing the type of immune response. Additional stimulation may be derived from IEL–IEL cross-talk, such as *via* OX40–XO40L interactions. IELs produce pro-inflammatory cytokines such as interferons (IFNs) and tumor necrosis factor (TNF), and cytotoxic factors such as Fas ligand (FasL) and granzymes, as well as antimicrobial peptides (AMPs) to contain the infection and contribute to wound healing and restoration of homeostasis by secreting growth factors such as KGF. Aberrant IEL activation and potentiation by cytokines might be involved in the development of chronic inflammation and IBD.

Intraepithelial lymphocyte maintenance and activation also critically relies on interactions between IECs and microorganisms. Myeloid differentiation primary response gene 88 (MyD88), the adapter protein used by many toll-like receptors (TLRs), interleukin (IL)-1R, and IL-18R activate the transcription factor nuclear factor-κB, is required for IEL maintenance *via* the production of IL-15 (65, 73) (Figure 2). IL-15 production signals *via* the type 1 transcription factor, Tbox expressed in T cells (Tbet) to maintain IEL precursors (51). TLR2 may be at least one of the pattern recognition receptors involved in IEL maintenance, *via* IL-15 induction, its absence resulting in marked reduction of intestinal IELs (74, 75). Although IL-15 may be induced by microorganisms, they may not be essential for its production as axenic mice have reportedly higher levels of *Il15* transcripts, and no differences in numbers of natural IELs were observed (51). Nucleotide-binding oligomerization domain-containing protein (NOD)2, an intracellular sensor for microbial products has also been shown to be important for IEL maintenance (76). IELs in NOD2-deficient mice show reduced proliferation and increased levels of apoptosis. Once again, NOD2 signaling, *via* recognition of gut microbiota, results in IL-15 production (Figure 2). Of interest, NOD2 is able to tune the signaling of TLR2 dose dependently (77). Although these results have been achieved using whole body knock outs for MyD88, TLR2, or NOD2, and therefore, the exact role of IECs remains to be determined, they indicate that microorganisms may play an important role in IEL maintenance.

### IEL Activation Status

Due to the positioning of IELs just underneath the single epithelial layer and their potential involvement in modulating intestinal pathology, the activation status of IELs is intensively studied. Transcriptional data of IELs foretells puzzling semi-activation of IELs that could enable them to deal with a broad range of pathologies quickly, with reduced requirement for immediate energy absorption and new gene expression (78). Unlike conventional CD8^+^ T cells, IELs express high levels of Tnfsf6 transcripts during steady state (42, 43), but do not express the encoding FasL protein on their surface until additional activation takes place (61). Despite their poised state and effector-like or T_EM_ cell characteristics, IELs do not contain transcripts for cytokines, which they secrete during conditions of inflammation (43). This suggests IELs require additional cues to initiate part of their effector function capacity.

Understanding the activation properties of IELs is essential to gain insight into mechanisms of local immunity and events associated with tolerance, chronic inflammation, and immunopathology. Intestinal mucosa resected from patients with IBD (79) or celiac disease (80) contains increased numbers of activated T cells, a hallmark of intestinal inflammatory disorders (81). Yet, in a chemically induced colitis model, dextran sulfate sodium, 2,4,6-trinitrobenzene sulfonic acid (TNBS) or T cell transfer colitis, and IELs were found to be protective (60, 76, 82–84). This raises questions regarding the role of IELs in the intestinal immune network, whether they can, at least in part, contribute to chronic inflammation and pathology, or if they have a more tolerogenic or regulatory role. Furthermore, although cytotoxicity and microbicidal activity are an important part of IEL activity, it is not clear if their potential to produce cytokines and chemokines can be tailored to the level or identity the microbial threat.

Transcriptional data also suggested that IELs are metabolically prepared for swift action. IELs, compared with memory CD8^+^ T cells, contain increased levels of mRNA for metabolic enzymes, especially those involved in the generation of fatty acids and cholesterol esters (42, 43). In line with the expression of CD69, IELs seem arrested in a semi-activated state. Yet, in stark contrast to effector cells, IELs survive for a considerable period of time. For example, murine skin IELs are generated only during embryogenesis, but can be found throughout adult life and into old age. The skin and intestinal epithelia are lipid-rich, but availability of other nutrients may be limited (85). This may explain why skin T_RM_ cells appear to use mitochondrial β-oxidation of exogenous lipids, mediated by intracellular transport proteins, including fatty-acid-binding protein-4 and -5, supporting their longevity and protective function (78). Similarly, natural IELs highly express surface molecules involved in lipid uptake, such as apolipoprotein E and low-density lipoprotein receptor (42). The increased presence of receptors and enzymes involved in lipid metabolism in IELs compared with conventional T cells suggests that altered metabolic processes may be involved in maintaining their poised activation status. However, it remains to be determined if the increase in lipid metabolism sets IELs apart or if it reflects their semi-activated status, since recently activated conventional T cells utilize the same pathways (86). Lipids are also required for the differentiation of CD8^+^ memory T cells, the formation of which requires metabolic reprogramming characterized by enhanced mitochondrial fatty-acid oxidation (87). Although, cell-intrinsic lipolysis is implicated in memory cell formation, suggesting the acquisition of fatty acids from the external environment is not critical, these lipids may have been obtained during the initial priming stage. These data imply that the metabolism of IELs reflects, in part, that of recently activated T cells and that lipolysis may be inhibited in IELs to arrest them in a poised activation status, thereby preventing progression to a quiescent memory status.

### Role for the TCR in IEL Activation

The understanding of the role of the TCR on IEL development, differentiation, homing, and activation has long been hampered by the absence of a known selective or activating ligand. Recent work has identified tissue-specific ligands expressed in the thymus, driving the development and homing of either murine skin IELs or intestinal TCRγδ IELs, not peptide-MHC or lipid-CD1 complexes, but the butyrophilin-like molecules skint-1 and butyrophilin-like 1 (Btnl1), respectively (88–90). The role of the TCRαβ expressed on natural CD8αα IELs remains unknown (91). The collective data from these studies strongly suggest that the TCRγδ is required for both thymic selection and imprinting of IEL identity as well as their maintenance in specific tissue niches. However, butyrophilins, part of the immunoglobulin superfamily [for detailed review see Rhodes et al. (92)], are, unlike MHC or CD1 molecules, not known to present ligands. Thus, it remains unclear if IELs are stimulated *via* their TCR or if they sense other cues, such as inflammatory, tissue damage or cell stress factors provided by IECs or accessory cells (93), or are under the influence of metabolic alterations as a result of cell damage or bacterial growth in the microenvironment which can enhance T_H_ cell subset differentiation (94).

Agonist-driven positive selection of IELs in the thymus suggests that mature IELs at the epithelial barriers could subsequently be activated by specific TCR ligands. IEL TCR activation may be achieved by cell surface receptors, such as non-classical MHC molecules (95, 96). The parameters required for an agonist to activate IELs upon conditions of inflammation or tissue damage exclude constitutively expressed surface molecules, such as skint-1 and Btnl1, unless a gradient reaching a critical activation threshold can be achieved. If an agonist able to activate IELs exists, it does not preclude direct IEL activation by microbial products such as observed for γδ T cells in both man and mouse (97–99). Such direct activation is commonly observed in conventional CD8^+^ T cells that have been pre-selected in the thymus and successfully primed and expanded in the periphery. Reactivation of memory CD8^+^ T cells is readily achieved by cytokines and TLR ligands, resulting in secretion of IFNγ from polyclonal T cells that bridge innate and adaptive immunity (100). Similarly, peripheral γδ T cells can be directly activated by TLR ligands and combinations of cytokines (99, 101).

Administration of anti-CD3ε antibodies, which directly stimulate the TCR signaling complex thereby bypassing TCR-specific ligation, has often been used as a proxy to stimulate IELs in mice. However, its systemic activity, due to indiscriminate total T cell activation in all tissues, results in “cytokine release syndrome,” increasing serum levels of IL-2, TNF, and IFNγ, and leading to intestinal phenotypes, such as diarrhea (102, 103). The small intestines from mice treated with anti-CD3 show increased epithelial ion transport, altered spontaneous muscle activity, and reduced IEC viability (104). The effect of anti-CD3 is rapid, with DNA fragmentation observed after 30 min in the areas most enriched with IELs, followed within hours by IEC shedding into the lumen (105). Similar effects on IEC viability were observed upon administration of anti-TCRγδ antibodies, but not with those stimulating TCRαβ (106). The effect on IEC shedding, however, was fully dependent on TNF receptor signaling and may not necessarily depend on IEL activation since conventional T cells can also secrete large amounts of TNF.

Following anti-CD3 stimulation, poised IELs acquire aspects of fully activated effector T cells with higher expression of CD44, Ly-6C, OX40, FasL, and CD25 and reduced expression of CD45RB protein, accompanied by expression of cytotoxic mediators as well as cytokine transcripts (61, 107). Effects of anti-CD3 on IEC viability appear to correlate well with the cytotoxic capacity of IELs, especially since release of granzyme B is observed upon anti-CD3 stimulation (62). However, DNA fragmentation is independent of the pore forming protein perforin (62). This suggests that IECs are non-specifically targeted by their proximity to activated T cells or by their susceptibility to soluble mediators. The accumulative data postulate that *in vivo* activation of IELs can at least in part be achieved *via* TCR ligation. IEL activity can have a major impact on intestinal physiology, altering electrolyte balance and IEC viability. However, their potential to damage IECs markedly contrast with the requirement to maintain an intact single cell intestinal barrier to efficiently protect the host and questions if TCR stimulation accurately recapitulates the physiological role of IELs. IEC–IEL bidirectional interactions are instrumental to maintain IELs, but it remains unknown if IECs directly contribute to IEL activation and, if they do, what the identities of the activating cues are?

### IEL Activation by Microbes

Commensal bacteria can invade tissues when opportunity arises. Such opportunities occur upon initial microbial invasion of new-borns before species-specific adaptive immunity has fully developed or when the host is immune compromised (108). Since, activating IELs may not require antigen processing or rely on presentation by MHC-like molecules, it remains possible that IELs recognize molecular patterns generated by bacterial non-peptide antigens or conserved unprocessed protein antigens produced by bacteria or released by epithelial cells upon damage or cell stress (109).

Invasion of pathogens or tissue damage could create the conditions for commensal microorganisms to invade the intestinal tissues. Innate immunity relies on the detection of highly conserved pathogen-associated molecular patterns (110). Receptors involved in the detection of invasion will respond to the microbial components present in both pathogen and commensal microorganisms. But it has become clear that not all microorganisms evoke a similar response. Indicators of viability, such as the presence of prokaryotic mRNA invoke a much stronger immune response (111). The balance of immunity and tolerance at the epithelial interphase is also illustrated by the production of IgA, the predominant antibody isotype critical at mucosal sites (112). IgA mainly coats pathogenic bacteria, which can confer colitis in axenic mice (113). IgA is a poor activator of the immune system; in line with the idea that strong immunity at mucosal sites is best avoided. IELs have been implicated in coordinating IgA response. TCRδ-deficient mice, harboring reduced IEL numbers, show reduced IgA levels in serum, saliva, and fecal samples. TCRδ-deficient mice also produce much lower levels of IgA antibodies upon oral immunization (114).

### Role for IECs in IEL Activation; Co-stimulation

A requirement for co-stimuli or T_H_ cell help is linked with the need for clonal expansion and differentiation, creating a significant delay in adaptive immunity. Immune surveillance by tissue-resident lymphocytes requires a swift response without prior cell expansion or differentiation, observed for γδ T cells and memory T cells, such as T_RM_ cells (27, 115). This is in line with the hypothesis that the IEL response primarily serves to contain a potential threat, not necessarily resulting in microbe eradication or the establishment of immunological memory, thereby limiting microbial or toxin dissemination and keeping the single cell barrier intact by avoiding intestinal pathology.

Homing of IELs to the small intestine seems consistent with oligoclonal activation by commonly encountered antigens. The poised activation status of IELs could ensure their rapid activation without the need for an array of instructive signals. IELs seem not to depend on signal 2, required for conventional T cell activation and protective immunity (116). CD28 as well as additional co-receptors, such as CD2 and CD5, appear reduced or absent from IELs (43, 117–119). Furthermore, expression of MHC molecules or the costimulatory B7 proteins on at least keratinocytes is not required for activation of skin IELs (97). However, since the triggering of IELs could contribute to immunopathology, their activation is likely controlled on several levels, such as by signals derived from inflammation or tissue damage. The absence of a requirement for classic costimulatory signals for IEL activation suggests that close interactions with IECs play a prominent role.

OX40 (CD134, TNFRSF4) is expressed by activated T cells controlling cell expansion (120), including IELs (121). Its expression correlates well with T cell activity observed in patients with IBD, active celiac disease, Crohn’s disease (CD), and ulcerative colitis (UC) (122, 123). *In vitro* activation of IELs with anti-CD3ε antibodies results in the expression of both OX40 and its ligand (OX40L) (121). Of note, OX40L is not expressed upon activation of conventional T cells. Additional ligation of OX40 seems to boost IEL activity and reduce the secretion of IL-10. This suggests that accumulation of IELs at sites of inflammation may alter their potential and that such co-stimulation may not necessarily depend on OX40L expression by IECs or myeloid cells (Figure 2).

Skin and intestinal IELs express the junctional adhesion-like molecule-1 (JAML-1), which provides co-stimulation upon ligation with the coxsackie-adenovirus receptor (CAR) (124, 125). JAML signaling results in cytokine production from skin IELs and may provide additional context for full IEL activation, presumably as response to infection or tissue damage. However, the latter requires its expression to be regulated upon insult or microbial invasion, which remains to be determined. Furthermore, its ligation by neutrophil-derived soluble JAML compromises intestinal barrier integrity and reduces wound repair through decreased IEC proliferation (126). Thus, the role of JAML—CAR in barrier defense remains to be clarified.

### Role for IECs in IEL Activation; Cytokines

Intestinal IELs can express receptors for TNF, leukemia inhibitory factor, thymic stromal lymphopoietin (TSLP), stem cell factor (SCF; c-Kit ligand), transforming growth factor (TGF)β, IL-12, IL-15, and IL-21 (43, 127). TGFβ, most likely derived from IECs upon microbial stimulation, is required to maintain natural CD8αα IELs and to induce CD103 expression. The absence of TGFβ or its receptor results in markedly reduced numbers of IELs, while over expression increased the IEL population (50). How TGFβ influences IEL activity remains unknown.

Interleukin-15 plays a central role in maintenance of natural IELs and emphasizes the close interactions between IECs and IELs (128–131). IL-15 is presented in *trans* to IELs by epithelial cells, in the thymus, skin, and the intestine, which express both the IL-15Rα and IL-15 (132). IL-15R signaling induces the expression of anti-apoptotic molecules, Bcl-2 and Bcl-xL by IELs (133). The production of IL-15 is regulated, at least in part, by contact with microbial components. MyD88- and TLR2-derived signals are required for IEL maintenance *via* the induction of IL-15 production (65, 73, 75). T_RM_ cells are similarly dependent on IL-15-mediated signals (134), whereby high levels of IL-15 can TCR-independently trigger CD8^+^ T cells to become cytotoxic (135, 136). Upon IEC damage, IL-15 production increases (65), as observed during celiac disease, and correlates strongly with IEL activity (133, 137). IL-15 stimulation of IELs results in increased IFNγ and TNF production, granzyme-dependent cytotoxicity, NK receptor expression, and increased survival (138). Of note, the increase in IL-15 production in conjunction with additional cues, such as retinoic acid can stimulate DCs, thereby inducing the secretion of pro-inflammatory factors and indirectly activate IELs. IL-15 induces the secretion of IL-21 by IELs, observed in celiac disease, which may be part of a self-sustaining feed-forward loop as observed in Th17 cells, enhancing IEL activation and cytotoxicity (139, 140).

Another important cytokine involved in T cell homeostasis is IL-7 (141). It is secreted by non-hematopoietic cells, especially thymic and IECs, with enhanced expression observed upon tissue damage (142–144). IEL development requires IL-7R signaling in the thymus, but local IL-7 expression by IECs can restore the γδIEL subset, not other γδ T cell subsets, suggesting extrathymic development or maturation of γδIELs may take place in the intestinal compartment (51, 142, 145). The use of acute IL-7 reporter mice indicates that production of IFNγ by T cells, such as IELs, can modulate the level of IL-7 and IL-15 produced by IECs, thereby regulating IEC homeostasis, absorptive function as well as the composition of the microbiota (144, 146). Vice versa, IEC derived IL-7 can regulate IEL survival and proliferation, particularly induced CD8αβ IELs (147). Overexpression of IL-7 results in lymphoid expansion and colitis (148).

Intestinal epithelial cells show a basal production of TSLP that is important for host protection during helminth infections (149). Similar to IL-15, TSLP receptor stimulation of CD8^+^ T cells enhances expression of Bcl-2 (150), and may play a role in IEL survival. TSLP seems to enhance type 2-mediated immunity (Figure 2). Upon TSLP encounter, IECs and DCs produce IL-10 and reduce IL-12 production, thereby reducing type 1 immunity (151). IELs are known to be able to secrete IFNγ and loss of intestinal integrity results in IEC-produced IL-12 (152), the prototypical driver of type 1 immunity. In the absence of TSLP, mice are more susceptible to colitis and have increased levels of IFNγ producing cells. *Salmonella typhimurium* infection increases the expression of SCF produced by IECs (153). Its receptor, c-Kit, is expressed by IELs (127). The absence of SCF results in marked reduction of IEL numbers in mice, while its presence seems to play a role in IEL activation (154). Whether SCF and TLSP act as instructive cues initiating divergent IEL activation profiles remains to be investigated.

### Containing Invasive Microbes

An ascending bacterial load exists from duodenum to jejunum and ileum, accumulating in very high numbers in the cecum and colon. Of note, IEL numbers are descending from duodenum to ileum, with few found in the colon (4). The causality of this striking inverted relationship remains unknown. IELs can produce antimicrobial factors and tissue repair factors in response to bacteria that penetrate the intestinal epithelium (60). IELs play an important role in the regulation and differentiation of epithelial cells at the base of the crypts (58, 155). IELs thereby help to preserve the integrity of damaged epithelial surface by providing the localized delivery of an epithelial cell growth factor (60). The mucosal protection afforded by IELs is of critical importance particularly during the first hours after bacterial exposure (156), in line with the hypothesis that IELs function to contain microbes upon invasion and initiate barrier repair thereby reducing immunopathology. With respect to pathogenic infections, in the majority studied, IELs offer protection against a wide variety of intestinal species, including *Eimeria vermiformis* (64, 157, 158), *Toxoplasma* (159, 160), *Encephalitozoon cuniculi* (161), Norovirus (107), and *Salmonella* (159, 160). Interestingly, infections with at least the pathogens *Salmonella* and *Toxoplasma* have indicated that IELs migrate to the site of infection and into the lateral intra-intestinal space (160), possibly initiating IEL–IEL co-stimulation (121). Collective release of preformed antimicrobial peptides (65, 156, 162) could directly contribute to microorganism containment and clearance (163).

Upon intestinal infectious challenges, protection and pathology are a result of the interplay between the microbes, the IELs, conventional T cells, and other immune cells. During *Eimeria* infection, IELs’ production of IFNγ and TNF is instrumental in protective immunity, and expression of junctional molecules to preserve epithelial barrier integrity (158). However, elevated IFNγ and TNF levels in the intestinal mucosa also contribute to the pro-inflammatory cascade involved in barrier disruption and pathology (164). γδIELs are able to reduce pathology and their absence exaggerates mucosal injury upon *Eimeria vermiformis* infection (157). The absence of αβ T cells results in reduced capacity to clear the parasite, in part compensated by the adoptive transfer of CD4^+^ T cells. In the absence of γδIELs, *Salmonella* or *Toxoplasma* infection result in increased microbe transmigration due to reduced epithelial barrier integrity. This increased transmigration leads to increased immunity mediated by conventional T cells (159). This indicates that IELs are not ultimately responsible for microbial clearance, but can modulate the initial response and recruitment of immune cells in order to moderate the risk of immunopathology.

Intraepithelial lymphocytes can contribute to viral immunity. Viral control depends largely on conventional T cells but, at least, αβIELs take part in viral clearance in the mucosa (165–167). Intestinal viral challenge, such as the non-enveloped RNA virus norovirus (MNV) present in many laboratory animal facilities, results in infection of IEC and myeloid cells (168). The infection can be controlled by IFNs, particularly IFNλ (169). Of note, the IFNλ (IFN type III) response seems to be operating particularly at epithelia barriers. This indicates that barrier immunity is kept local, to avoid systemic responses in which type I IFNs play a dominant role (170, 171). IELs, upon stimulation with plate-bound anti-CD3, can transcribe IFN genes, type I, II, and III, and the supernatant of *in vitro* activated IELs reduces viral infection (107, 172). *In vivo* anti-CD3 administration as well as culture supernatant from activated IELs results in IFN type I/III receptor-dependent expression of IFN responsive genes in intestinal IECs. Administration of anti-CD3 antibodies before intestinal viral challenge with murine norovirus can reduce viral load (107). However, due to the polyclonal stimulation of all T-lymphocytes, it remains unclear what contribution IELs provide and which properties may be uniquely attributed to them. It remains unknown if IELs are stimulated upon intestinal viral invasion and if so, how such invasion would enable the activation of IELs.

Besides microbicidal and cytotoxic activity, IELs produce cytokines and chemokines. Some chemokine transcripts are already present in IELs under steady state conditions, such as CCL5 and XCL1, but not those encoding for cytokines, such as IFNγ and TNF (43). This suggests that the recruitment of additional immune cells and release of these powerful cytokines and other chemoattractants such as CXCL1, CXCL2, and CXCL9 (65), might be delayed compared with cytotoxicity. The clearance of pathogens and the instigation of immunological memory involve careful orchestration of various cellular components. At epithelial sites, pathogen containment by innate-like T-lymphocytes may precede recruitment of myeloid cells and subsequent involvement of the adoptive arm of the immune response.

### IELs in IBDs

The initiation of an immune response is not taken lightly, especially at the intestinal barrier. When immune activation does take place, pathogen clearance, pathology, and the need to maintain tissue integrity and its repair are offset. The consequences of microbial invasion or aberrant immunity can be severe (81). IBD can affect any part of the intestine and present with extra-intestinal manifestations. Despite advances in IBD understanding, the cause(s) and mechanism(s) remain unknown and disease incidence is increasing with changes in the environment and life style likely making a contribution (173). Disease occurrence and severity are further compounded by diet, dehydration, and antibiotic use (174) as well as age (175).

There are indications that IBD results from alterations in innate immunity resulting in excessive adaptive immune activation (176, 177). IBD seems to result from immune-genetic predispositions and environmental factors, especially a dysregulated response to microorganisms (178). The presence of micro-infections or patches of affected intestinal tissue, associated with bacterial presence, next to seemingly unaffected tissue suggest localized immune activation or inefficient immune resolution (179). Several immunologic and histopathologic features of IBD, such as the presence of activated T cells secreting IFNγ and IL-17 and immunopathology (180), can be explained as a defect in mucosal immune regulation and as a consequence of persistent mucosal T cell activation (181). Several studies have shown that IL-12 production is increased in inflammatory lesions of patients with CD (182). In line with activated T cells, UC patients have an increased level of IL-7 and IL- 15, which may perpetrate additional T cell activation (183, 184).

There is limited data for a role of IELs in IBD, either disease enhancing or reducing. Accumulation of γδIELs in inflamed areas of IBD patients have been reported, and associated with increased levels of IL-15 and TSLP (133, 151). Although identifying lamina propria migrated γδ T cells and γδIELs is difficult, these cells constitutively produce IFNγ in patients suffering from either CD or UC (185). It remains to be determined if reduced numbers of IELs contribute to increased susceptibility to inflammation or are a consequence of ongoing inflammation, such as due to an increase in αβ effector T cells or activation-related cell death. Murine studies have demonstrated potential protective roles for γδIELs in intestinal inflammation as well as IBD models (59, 82, 186), but others have suggested that IEL expansion and activation can exacerbate the progression of colitis (187, 188). Lesions in IBD are mainly found in areas of reduced IEL density and highest load of bacteria, the colon, and ileum (178). The numbers of IELs correlate inversely with disease severity, and IEL numbers are restored to levels observed in healthy controls upon treatment with anti-TNF (189). This indicates vulnerability at those sites with altered immune surveillance and intense bacteria–IEC interactions, in line with the presence of adherent-invasive *Escherichia coli* at IBD lesions (179).

Loss-of-function mutations of NOD2 are strongly associated with CD (190–193). This correlates with the loss of IELs seen in mice deficient for NOD2 (76), but detailed insights are lacking and NOD2 plays an important role in other cell populations such as DCs, able to influence type 1 and type 3 immunity (194). Reduced proportions of γδIELs at the intestinal mucosa of CD patients suggested a protective role for these cells (189, 195, 196). IELs show increased activity at inflammatory sites in IBD patients, secreting IFNγ and TNF (189). Furthermore, IELs may enhance the production of IFNγ in the human colon (197). Nevertheless, murine IELs have been shown to be able to reduce the production of IFNγ production by conventional CD4 T cells, indicating their capacity to reduce type 1 immunity (198). In line with IELs forming an important part of the first line of defense is the increased susceptibility to infection of CD patients, which have reduced IEL numbers, with the intracellular parasite microsporidia (199). Unfortunately, the data on a role for IELs in preventing or reducing susceptibility to IBD remain inconclusive. The majority of IELs are found in the small intestine, a site not easily accessible, and IELs as a population of human T-lymphocytes are not well defined with respect to their identity and location. Often γδ T cells, such as those found in the circulation, are used as a proxy for IELs. However, murine studies and data on human TCRδ-chain usage have shown IELs to be very different from γδ T cells found in other tissues [(55, 59) #1587].

## Conclusion

Intraepithelial lymphocytes are an integral part of the epithelial barrier. They do not exist as isolated cells monitoring the front line, but have close bidirectional interactions with IECs and possibly other immune cells. This close interaction enables the reception and provision of signals at very close range to maintain epithelial integrity. This interaction may be crucial in initiating local repair and containing low level of microbial invasion. Localized cues of potentially low dose would enable the activation of poised IELs without necessarily alerting the adaptive immune system. The single cell epithelial barrier is under constant threat of assault. Containing such threats locally with minimal immune activation is of great benefit to limit immunopathology and maintain optimal nutrient uptake.

How IELs, displaying many characteristics of effector T cells, are maintained in a poised state remains poorly understood. Differences identified in metabolic pathways may reflect their partial activation status or indicate differential metabolic wiring of IELs. The cues that enable full IEL activation remain ill-defined and the activity of IELs, the existence of different modes of action, are unknown. With technological advantages, such as multicolor flow cytometry and microscopy new players at the mucosal sites have been identified. This may now help us to unravel the complex multiplayer immune surveillance network of the mucosal immune response with real potential to discover novel targets for therapies to alleviate or even cure the different forms of IBD and celiac disease.

## Author Contributions

All authors wrote the review and contributed to the figures.

## Conflict of Interest Statement

The authors declare that the research was conducted in the absence of any commercial or financial relationships that could be construed as a potential conflict of interest.

## References

[1] Cieza RJ Cao AT Cong Y Torres AG Immunomodulation for gastrointestinal infections. Expert Rev Anti Infect Ther (2012) 10(3):391–400. 10.1586/eri.11.176 22397571 PMC3319044

[2] Medzhitov R Janeway C Jr Innate immunity. N Engl J Med (2000) 343(5):338–44. 10.1056/NEJM200008033430506 10922424

[3] Guilliams M Ginhoux F Jakubzick C Naik SH Onai N Schraml BU Dendritic cells, monocytes and macrophages: a unified nomenclature based on ontogeny. Nat Rev Immunol (2014) 14(8):571–8. 10.1038/nri3712 25033907 PMC4638219

[4] Mowat AM Agace WW Regional specialization within the intestinal immune system. Nat Rev Immunol (2014) 14(10):667–85. 10.1038/nri3738 25234148

[5] Veldhoen M. The role of T helper subsets in autoimmunity and allergy. Curr Opin Immunol (2009) 21(6):606–11. 10.1016/j.coi.2009.07.009 19683910

[6] Brucklacher-Waldert V Carr EJ Linterman MA Veldhoen M. Cellular plasticity of CD4+ T cells in the intestine. Front Immunol (2014) 5:488. 10.3389/fimmu.2014.00488 25339956 PMC4188036

[7] Ohnmacht C Park JH Cording S Wing JB Atarashi K Obata Y Mucosal immunology. The microbiota regulates type 2 immunity through RORgammat(+) T cells. Science (2015) 349(6251):989–93. 10.1126/science.aac4263 26160380

[8] Laidlaw BJ Craft JE Kaech SM The multifaceted role of CD4(+) T cells in CD8(+) T cell memory. Nat Rev Immunol (2016) 16(2):102–11. 10.1038/nri.2015.10 26781939 PMC4860014

[9] Cheroutre H Lambolez F Mucida D. The light and dark sides of intestinal intraepithelial lymphocytes. Nat Rev Immunol (2011) 11(7):445–56. 10.1038/nri3007 21681197 PMC3140792

[10] Cepek KL Shaw SK Parker CM Russell GJ Morrow JS Rimm DL Adhesion between epithelial cells and T lymphocytes mediated by E-cadherin and the alpha E beta 7 integrin. Nature (1994) 372(6502):190–3. 10.1038/372190a0 7969453

[11] Beagley KW Fujihashi K Lagoo AS Lagoo-Deenadaylan S Black CA Murray AM Differences in intraepithelial lymphocyte T cell subsets isolated from murine small versus large intestine. J Immunol (1995) 154(11):5611–9. 7751614

[12] Mysorekar IU Lorenz RG Gordon JI. A gnotobiotic transgenic mouse model for studying interactions between small intestinal enterocytes and intraepithelial lymphocytes. J Biol Chem (2002) 277(40):37811–9. 10.1074/jbc.M205300200 12138109

[13] Qi H Kastenmuller W Germain RN. Spatiotemporal basis of innate and adaptive immunity in secondary lymphoid tissue. Annu Rev Cell Dev Biol (2014) 30:141–67. 10.1146/annurev-cellbio-100913-013254 25150013

[14] Jung S Unutmaz D Wong P Sano G De los Santos K Sparwasser T In vivo depletion of CD11c(+) dendritic cells abrogates priming of CD8(+) T cells by exogenous cell-associated antigens. Immunity (2002) 17(2):211–20. 10.1016/S1074-7613(02)00365-5 12196292 PMC3689299

[15] Mescher MF Curtsinger JM Agarwal P Casey KA Gerner M Hammerbeck CD Signals required for programming effector and memory development by CD8+ T cells. Immunol Rev (2006) 211:81–92. 10.1111/j.0105-2896.2006.00382.x 16824119

[16] Chen L Flies DB. Molecular mechanisms of T cell co-stimulation and co-inhibition. Nat Rev Immunol (2013) 13(4):227–42. 10.1038/nri3405 23470321 PMC3786574

[17] Knickelbein JE Khanna KM Yee MB Baty CJ Kinchington PR Hendricks RL. Noncytotoxic lytic granule-mediated CD8+ T cell inhibition of HSV-1 reactivation from neuronal latency. Science (2008) 322(5899):268–71. 10.1126/science.1164164 18845757 PMC2680315

[18] Masopust D Vezys V Marzo AL Lefrancois L. Preferential localization of effector memory cells in nonlymphoid tissue. Science (2001) 291(5512):2413–7. 10.1126/science.1058867 11264538

[19] Hildeman DA Zhu Y Mitchell TC Bouillet P Strasser A Kappler J Activated T cell death in vivo mediated by proapoptotic bcl-2 family member bim. Immunity (2002) 16(6):759–67. 10.1016/S1074-7613(02)00322-9 12121658

[20] Tanchot C Guillaume S Delon J Bourgeois C Franzke A Sarukhan A Modifications of CD8+ T cell function during in vivo memory or tolerance induction. Immunity (1998) 8(5):581–90. 10.1016/S1074-7613(00)80563-4 9620679

[21] Veiga-Fernandes H Walter U Bourgeois C McLean A Rocha B Response of naive and memory CD8+ T cells to antigen stimulation in vivo. Nat Immunol (2000) 1(1):47–53. 10.1038/76907 10881174

[22] Reinhardt RL Khoruts A Merica R Zell T Jenkins MK. Visualizing the generation of memory CD4 T cells in the whole body. Nature (2001) 410(6824):101–5. 10.1038/35065111 11242050

[23] Whitmire JK Eam B Whitton JL. Tentative T cells: memory cells are quick to respond, but slow to divide. PLoS Pathog (2008) 4(4):e1000041. 10.1371/journal.ppat.1000041 18404208 PMC2275797

[24] Hamann D Baars PA Rep MH Hooibrink B Kerkhof-Garde SR Klein MR Phenotypic and functional separation of memory and effector human CD8+ T cells. J Exp Med (1997) 186(9):1407–18. 10.1084/jem.186.9.1407 9348298 PMC2199103

[25] Sallusto F Lenig D Forster R Lipp M Lanzavecchia A. Two subsets of memory T lymphocytes with distinct homing potentials and effector functions. Nature (1999) 401(6754):708–12. 10.1038/44385 10537110

[26] Schon MP Arya A Murphy EA Adams CM Strauch UG Agace WW Mucosal T lymphocyte numbers are selectively reduced in integrin alpha E (CD103)-deficient mice. J Immunol (1999) 162(11):6641–9. 10352281

[27] Gebhardt T Wakim LM Eidsmo L Reading PC Heath WR Carbone FR. Memory T cells in nonlymphoid tissue that provide enhanced local immunity during infection with herpes simplex virus. Nat Immunol (2009) 10(5):524–30. 10.1038/ni.1718 19305395

[28] Casey KA Fraser KA Schenkel JM Moran A Abt MC Beura LK Antigen-independent differentiation and maintenance of effector-like resident memory T cells in tissues. J Immunol (2012) 188(10):4866–75. 10.4049/jimmunol.1200402 22504644 PMC3345065

[29] Mackay LK Rahimpour A Ma JZ Collins N Stock AT Hafon ML The developmental pathway for CD103(+)CD8+ tissue-resident memory T cells of skin. Nat Immunol (2013) 14(12):1294–301. 10.1038/ni.2744 24162776

[30] Stary G Olive A Radovic-Moreno AF Gondek D Alvarez D Basto PA VACCINES. A mucosal vaccine against *Chlamydia trachomatis* generates two waves of protective memory T cells. Science (2015) 348(6241):aaa8205 10.1126/science.aaa8205 26089520 PMC4605428

[31] Springer TA Traffic signals for lymphocyte recirculation and leukocyte emigration: the multistep paradigm. Cell (1994) 76(2):301–14. 10.1016/0092-8674(94)90337-9 7507411

[32] Forster R Schubel A Breitfeld D Kremmer E Renner-Muller I Wolf E CCR7 coordinates the primary immune response by establishing functional microenvironments in secondary lymphoid organs. Cell (1999) 99(1):23–33. 10.1016/S0092-8674(00)80059-8 10520991

[33] Bromley SK Thomas SY Luster AD. Chemokine receptor CCR7 guides T cell exit from peripheral tissues and entry into afferent lymphatics. Nat Immunol (2005) 6(9):895–901. 10.1038/ni1240 16116469

[34] Debes GF Arnold CN Young AJ Krautwald S Lipp M Hay JB Chemokine receptor CCR7 required for T lymphocyte exit from peripheral tissues. Nat Immunol (2005) 6(9):889–94. 10.1038/ni1238 16116468 PMC2144916

[35] Masopust D Choo D Vezys V Wherry EJ Duraiswamy J Akondy R Dynamic T cell migration program provides resident memory within intestinal epithelium. J Exp Med (2010) 207(3):553–64. 10.1084/jem.20090858 20156972 PMC2839151

[36] Jiang X Clark RA Liu L Wagers AJ Fuhlbrigge RC Kupper TS. Skin infection generates non-migratory memory CD8+ T(RM) cells providing global skin immunity. Nature (2012) 483(7388):227–31. 10.1038/nature10851 22388819 PMC3437663

[37] Schenkel JM Fraser KA Vezys V Masopust D Sensing and alarm function of resident memory CD8(+) T cells. Nat Immunol (2013) 14(5):509–13. 10.1038/ni.2568 23542740 PMC3631432

[38] Wakim LM Waithman J van Rooijen N Heath WR Carbone FR. Dendritic cell-induced memory T cell activation in nonlymphoid tissues. Science (2008) 319(5860):198–202. 10.1126/science.1151869 18187654

[39] Zaid A Mackay LK Rahimpour A Braun A Veldhoen M Carbone FR Persistence of skin-resident memory T cells within an epidermal niche. Proc Natl Acad Sci U S A (2014) 111(14):5307–12. 10.1073/pnas.1322292111 24706879 PMC3986170

[40] Masopust D Schenkel JM The integration of T cell migration, differentiation and function. Nat Rev Immunol (2013) 13(5):309–20. 10.1038/nri3442 23598650

[41] Steinert EM Schenkel JM Fraser KA Beura LK Manlove LS Igyarto BZ Quantifying memory CD8 T cells reveals regionalization of immunosurveillance. Cell (2015) 161(4):737–49. 10.1016/j.cell.2015.03.031 25957682 PMC4426972

[42] Fahrer AM Konigshofer Y Kerr EM Ghandour G Mack DH Davis MM Attributes of gammadelta intraepithelial lymphocytes as suggested by their transcriptional profile. Proc Natl Acad Sci U S A (2001) 98(18):10261–6. 10.1073/pnas.171320798 11526237 PMC56949

[43] Shires J Theodoridis E Hayday AC Biological insights into TCRgammadelta+ and TCRalphabeta+ intraepithelial lymphocytes provided by serial analysis of gene expression (SAGE). Immunity (2001) 15(3):419–34. 10.1016/S1074-7613(01)00192-3 11567632

[44] Suzuki H Jeong K II Doi K. Age-related changes in the regional variations in the number and subsets of intraepithelial lymphocytes in mouse small intestine. Dev Comp Immunol (2002) 26(6):589–95. 10.1016/S0145-305X(02)00004-6 12031418

[45] Rocha B Vassalli P Guy-Grand D. Thymic and extrathymic origins of gut intraepithelial lymphocyte populations in mice. J Exp Med (1994) 180(2):681–6. 10.1084/jem.180.2.681 8046341 PMC2191614

[46] Leishman AJ Gapin L Capone M Palmer E MacDonald HR Kronenberg M Precursors of functional MHC class I- or class II-restricted CD8alphaalpha(+) T cells are positively selected in the thymus by agonist self-peptides. Immunity (2002) 16(3):355–64. 10.1016/S1074-7613(02)00284-4 11911821

[47] Eberl G Littman DR. Thymic origin of intestinal alphabeta T cells revealed by fate mapping of RORgammat+ cells. Science (2004) 305(5681):248–51. 10.1126/science.1096472 15247480

[48] Yamagata T Mathis D Benoist C. Self-reactivity in thymic double-positive cells commits cells to a CD8 alpha alpha lineage with characteristics of innate immune cells. Nat Immunol (2004) 5(6):597–605. 10.1038/ni1070 15133507

[49] Gangadharan D Lambolez F Attinger A Wang-Zhu Y Sullivan BA Cheroutre H Identification of pre- and postselection TCRalphabeta+ intraepithelial lymphocyte precursors in the thymus. Immunity (2006) 25(4):631–41. 10.1016/j.immuni.2006.08.018 17045820

[50] Konkel JE Maruyama T Carpenter AC Xiong Y Zamarron BF Hall BE Control of the development of CD8alphaalpha+ intestinal intraepithelial lymphocytes by TGF-beta. Nat Immunol (2011) 12(4):312–9. 10.1038/ni.1997 21297643 PMC3062738

[51] Klose CS Blatz K d’Hargues Y Hernandez PP Kofoed-Nielsen M Ripka JF The transcription factor T-bet is induced by IL-15 and thymic agonist selection and controls CD8alphaalpha(+) intraepithelial lymphocyte development. Immunity (2014) 41(2):230–43. 10.1016/j.immuni.2014.06.018 25148024

[52] Leishman AJ Naidenko OV Attinger A Koning F Lena CJ Xiong Y T cell responses modulated through interaction between CD8alphaalpha and the nonclassical MHC class I molecule, TL. Science (2001) 294(5548):1936–9. 10.1126/science.1063564 11729321

[53] Goodman T Lefrancois L Expression of the gamma-delta T-cell receptor on intestinal CD8+ intraepithelial lymphocytes. Nature (1988) 333(6176):855–8. 10.1038/333855a0 2968521

[54] Deusch K Luling F Reich K Classen M Wagner H Pfeffer K. A major fraction of human intraepithelial lymphocytes simultaneously expresses the gamma/delta T cell receptor, the CD8 accessory molecule and preferentially uses the V delta 1 gene segment. Eur J Immunol (1991) 21(4):1053–9. 10.1002/eji.1830210429 1826884

[55] Dunne MR Elliott L Hussey S Mahmud N Kelly J Doherty DG Persistent changes in circulating and intestinal gammadelta T cell subsets, invariant natural killer T cells and mucosal-associated invariant T cells in children and adults with coeliac disease. PLoS One (2013) 8(10):e76008 10.1371/journal.pone.0076008 24124528 PMC3790827

[56] Brandes M Willimann K Bioley G Levy N Eberl M Luo M Cross-presenting human gammadelta T cells induce robust CD8+ alphabeta T cell responses. Proc Natl Acad Sci U S A (2009) 106(7):2307–12. 10.1073/pnas.0810059106 19171897 PMC2650152

[57] Chennupati V Worbs T Liu X Malinarich FH Schmitz S Haas JD Intra- and intercompartmental movement of gammadelta T cells: intestinal intraepithelial and peripheral gammadelta T cells represent exclusive nonoverlapping populations with distinct migration characteristics. J Immunol (2010) 185(9):5160–8. 10.4049/jimmunol.1001652 20870939

[58] Komano H Fujiura Y Kawaguchi M Matsumoto S Hashimoto Y Obana S Homeostatic regulation of intestinal epithelia by intraepithelial gamma delta T cells. Proc Natl Acad Sci U S A (1995) 92(13):6147–51. 10.1073/pnas.92.13.6147 7597094 PMC41659

[59] Li Y Innocentin S Withers DR Roberts NA Gallagher AR Grigorieva EF Exogenous stimuli maintain intraepithelial lymphocytes via aryl hydrocarbon receptor activation. Cell (2011) 147(3):629–40. 10.1016/j.cell.2011.09.025 21999944

[60] Chen Y Chou K Fuchs E Havran WL Boismenu R. Protection of the intestinal mucosa by intraepithelial gamma delta T cells. Proc Natl Acad Sci U S A (2002) 99(22):14338–43. 10.1073/pnas.212290499 12376619 PMC137885

[61] Wang HC Zhou Q Dragoo J Klein JR. Most murine CD8+ intestinal intraepithelial lymphocytes are partially but not fully activated T cells. J Immunol (2002) 169(9):4717–22. 10.4049/jimmunol.169.9.4717 12391179

[62] Ogata M Ota Y Matsutani T Nanno M Suzuki R Itoh T. Granzyme B-dependent and perforin-independent DNA fragmentation in intestinal epithelial cells induced by anti-CD3 mAb-activated intra-epithelial lymphocytes. Cell Tissue Res (2013) 352(2):287–300. 10.1007/s00441-012-1549-7 23361111

[63] Yang H Antony PA Wildhaber BE Teitelbaum DH Intestinal intraepithelial lymphocyte gamma delta-T cell-derived keratinocyte growth factor modulates epithelial growth in the mouse. J Immunol (2004) 172(7):4151–8. 10.4049/jimmunol.172.7.4151 15034027

[64] Smith AL Hayday AC. An alphabeta T-cell-independent immunoprotective response towards gut coccidia is supported by gammadelta cells. Immunology (2000) 101(3):325–32. 10.1046/j.1365-2567.2000.00122.x 11106935 PMC2327095

[65] Ismail AS Behrendt CL Hooper LV. Reciprocal interactions between commensal bacteria and gamma delta intraepithelial lymphocytes during mucosal injury. J Immunol (2009) 182(5):3047–54. 10.4049/jimmunol.0802705 19234201 PMC2763635

[66] Cai SF Fehniger TA Cao X Mayer JC Brune JD French AR Differential expression of granzyme B and C in murine cytotoxic lymphocytes. J Immunol (2009) 182(10):6287–97. 10.4049/jimmunol.0804333 19414782 PMC2714542

[67] Lambolez F Arcangeli ML Joret AM Pasqualetto V Cordier C Di Santo JP The thymus exports long-lived fully committed T cell precursors that can colonize primary lymphoid organs. Nat Immunol (2006) 7(1):76–82. 10.1038/ni1293 16341216

[68] Staton TL Habtezion A Winslow MM Sato T Love PE Butcher EC. CD8+ recent thymic emigrants home to and efficiently repopulate the small intestine epithelium. Nat Immunol (2006) 7(5):482–8. 10.1038/ni1319 16582913

[69] Lefrancois L Goodman T. In vivo modulation of cytolytic activity and Thy-1 expression in TCR-gamma delta+ intraepithelial lymphocytes. Science (1989) 243(4899):1716–8. 10.1126/science.2564701 2564701

[70] Kawaguchi M Nanno M Umesaki Y Matsumoto S Okada Y Cai Z Cytolytic activity of intestinal intraepithelial lymphocytes in germ-free mice is strain dependent and determined by T cells expressing gamma delta T-cell antigen receptors. Proc Natl Acad Sci U S A (1993) 90(18):8591–4. 10.1073/pnas.90.18.8591 8378333 PMC47403

[71] Stange J Veldhoen M. The aryl hydrocarbon receptor in innate T cell immunity. Semin Immunopathol (2013) 35(6):645–55. 10.1007/s00281-013-0389-1 24030775

[72] Moura-Alves P Fae K Houthuys E Dorhoi A Kreuchwig A Furkert J AhR sensing of bacterial pigments regulates antibacterial defence. Nature (2014) 512(7515):387–92. 10.1038/nature13684 25119038

[73] Yu Q Tang C Xun S Yajima T Takeda K Yoshikai Y. MyD88-dependent signaling for IL-15 production plays an important role in maintenance of CD8 alpha alpha TCR alpha beta and TCR gamma delta intestinal intraepithelial lymphocytes. J Immunol (2006) 176(10):6180–5. 10.4049/jimmunol.176.10.6180 16670327

[74] Rakoff-Nahoum S Paglino J Eslami-Varzaneh F Edberg S Medzhitov R. Recognition of commensal microflora by toll-like receptors is required for intestinal homeostasis. Cell (2004) 118(2):229–41. 10.1016/j.cell.2004.07.002 15260992

[75] Qiu Y Pu A Zheng H Liu M Chen W Wang W TLR2-dependent signaling for IL-15 production is essential for the homeostasis of intestinal intraepithelial lymphocytes. Mediators Inflamm (2016) 2016:4281865. 10.1155/2016/4281865 27563173 PMC4983668

[76] Jiang W Wang X Zeng B Liu L Tardivel A Wei H Recognition of gut microbiota by NOD2 is essential for the homeostasis of intestinal intraepithelial lymphocytes. J Exp Med (2013) 210(11):2465–76. 10.1084/jem.20122490 24062413 PMC3804938

[77] Borm ME van Bodegraven AA Mulder CJ Kraal G Bouma G. The effect of NOD2 activation on TLR2-mediated cytokine responses is dependent on activation dose and NOD2 genotype. Genes Immun (2008) 9(3):274–8. 10.1038/gene.2008.9 18340358

[78] Pan Y Tian T Park CO Lofftus SY Mei S Liu X Survival of tissue-resident memory T cells requires exogenous lipid uptake and metabolism. Nature (2017) 543(7644):252–6. 10.1038/nature21379 28219080 PMC5509051

[79] Burgio VL Fais S Boirivant M Perrone A Pallone F. Peripheral monocyte and naive T-cell recruitment and activation in Crohn’s disease. Gastroenterology (1995) 109(4):1029–38. 10.1016/0016-5085(95)90560-X 7557067

[80] Maiuri L Picarelli A Boirivant M Coletta S Mazzilli MC De Vincenzi M Definition of the initial immunologic modifications upon in vitro gliadin challenge in the small intestine of celiac patients. Gastroenterology (1996) 110(5):1368–78. 10.1053/gast.1996.v110.pm8613040 8613040

[81] Fonseca-Camarillo G Yamamoto-Furusho JK. Immunoregulatory pathways involved in inflammatory bowel disease. Inflamm Bowel Dis (2015) 21(9):2188–93. 10.1097/MIB.0000000000000477 26111210

[82] Hoffmann JC Peters K Henschke S Herrmann B Pfister K Westermann J Role of T lymphocytes in rat 2,4,6-trinitrobenzene sulphonic acid (TNBS) induced colitis: increased mortality after gammadelta T cell depletion and no effect of alphabeta T cell depletion. Gut (2001) 48(4):489–95. 10.1136/gut.48.4.489 11247892 PMC1728226

[83] Poussier P Ning T Banerjee D Julius M. A unique subset of self-specific intraintestinal T cells maintains gut integrity. J Exp Med (2002) 195(11):1491–7. 10.1084/jem.20011793 12045247 PMC2193537

[84] Tsuchiya T Fukuda S Hamada H Nakamura A Kohama Y Ishikawa H Role of gamma delta T cells in the inflammatory response of experimental colitis mice. J Immunol (2003) 171(10):5507–13. 10.4049/jimmunol.171.10.5507 14607957

[85] Zhang Y Li Q Rao E Sun Y Grossmann ME Morris RJ Epidermal fatty acid binding protein promotes skin inflammation induced by high-fat diet. Immunity (2015) 42(5):953–64. 10.1016/j.immuni.2015.04.016 25992864 PMC4440244

[86] van der Windt GJ Everts B Chang CH Curtis JD Freitas TC Amiel E Mitochondrial respiratory capacity is a critical regulator of CD8+ T cell memory development. Immunity (2012) 36(1):68–78. 10.1016/j.immuni.2011.12.007 22206904 PMC3269311

[87] O’Sullivan D van der Windt GJ Huang SC Curtis JD Chang CH Buck MD Memory CD8(+) T cells use cell-intrinsic lipolysis to support the metabolic programming necessary for development. Immunity (2014) 41(1):75–88. 10.1016/j.immuni.2014.06.005 25001241 PMC4120664

[88] Barbee SD Woodward MJ Turchinovich G Mention JJ Lewis JM Boyden LM Skint-1 is a highly specific, unique selecting component for epidermal T cells. Proc Natl Acad Sci U S A (2011) 108(8):3330–5. 10.1073/pnas.1010890108 21300860 PMC3044407

[89] Turchinovich G Hayday AC Skint-1 identifies a common molecular mechanism for the development of interferon-gamma-secreting versus interleukin-17-secreting gammadelta T cells. Immunity (2011) 35(1):59–68. 10.1016/j.immuni.2011.04.018 21737317

[90] Di Marco Barros R Roberts NA Dart RJ Vantourout P Jandke A Nussbaumer O Epithelia use butyrophilin-like molecules to shape organ-specific gammadelta T cell compartments. Cell (2016) 167(1):203–218e217. 10.1016/j.cell.2016.08.030 27641500 PMC5037318

[91] Park SH Guy-Grand D Lemonnier FA Wang CR Bendelac A Jabri B. Selection and expansion of CD8alpha/alpha(1) T cell receptor alpha/beta(1) intestinal intraepithelial lymphocytes in the absence of both classical major histocompatibility complex class I and nonclassical CD1 molecules. J Exp Med (1999) 190(6):885–90. 10.1084/jem.190.6.885 10499927 PMC2195634

[92] Rhodes DA Reith W Trowsdale J. Regulation of immunity by butyrophilins. Annu Rev Immunol (2016) 34:151–72. 10.1146/annurev-immunol-041015-055435 26772212

[93] Hayday AC Gammadelta T cells and the lymphoid stress-surveillance response. Immunity (2009) 31(2):184–96. 10.1016/j.immuni.2009.08.006 19699170

[94] Brucklacher-Waldert V Ferreira C Stebegg M Fesneau F Innocentin S Marie JC Cellular stress in context of an inflammatory environment supports TGFβ independent T helper-17 differentiation. Cell Rep (2017) 19(11). 10.1016/j.celrep.2017.05.052 PMC5483510 28614720

[95] Adams EJ Chien YH Garcia KC. Structure of a gammadelta T cell receptor in complex with the nonclassical MHC T22. Science (2005) 308(5719):227–31. 10.1126/science.1106885 15821084

[96] Adams EJ Strop P Shin S Chien YH Garcia KC. An autonomous CDR3delta is sufficient for recognition of the nonclassical MHC class I molecules T10 and T22 by gammadelta T cells. Nat Immunol (2008) 9(7):777–84. 10.1038/ni.1620 18516039 PMC2768525

[97] Hayday AC. [gamma][delta] cells: a right time and a right place for a conserved third way of protection. Annu Rev Immunol (2000) 18:975–1026. 10.1146/annurev.immunol.18.1.975 10837080

[98] Scotet E Martinez LO Grant E Barbaras R Jeno P Guiraud M Tumor recognition following Vgamma9Vdelta2 T cell receptor interactions with a surface F1-ATPase-related structure and apolipoprotein A-I. Immunity (2005) 22(1):71–80. 10.1016/j.immuni.2004.11.012 15664160

[99] Martin B Hirota K Cua DJ Stockinger B Veldhoen M. Interleukin-17-producing gammadelta T cells selectively expand in response to pathogen products and environmental signals. Immunity (2009) 31(2):321–30. 10.1016/j.immuni.2009.06.020 19682928

[100] Leggat JA Gibbons DL Haque SF Smith AL Wells JW Choy K Innate responsiveness of CD8 memory T-cell populations nonspecifically inhibits allergic sensitization. J Allergy Clin Immunol (2008) 122(5):e1014 10.1016/j.jaci.2008.08.011 PMC3389734 18804851

[101] Sutton CE Lalor SJ Sweeney CM Brereton CF Lavelle EC Mills KH. Interleukin-1 and IL-23 induce innate IL-17 production from gammadelta T cells, amplifying Th17 responses and autoimmunity. Immunity (2009) 31(2):331–41. 10.1016/j.immuni.2009.08.001 19682929

[102] Ferran C Sheehan K Dy M Schreiber R Merite S Landais P Cytokine-related syndrome following injection of anti-CD3 monoclonal antibody: further evidence for transient in vivo T cell activation. Eur J Immunol (1990) 20(3):509–15. 10.1002/eji.1830200308 2138557

[103] Finck BK Yung CM Carteron NL Wofsy D The role of T-cell subsets in the response to anti-CD3 monoclonal antibodies. Clin Immunol Immunopathol (1992) 65(3):234–41. 10.1016/0090-1229(92)90152-E 1360341

[104] Radojevic N McKay DM Merger M Vallance BA Collins SM Croitoru K. Characterization of enteric functional changes evoked by in vivo anti-CD3 T cell activation. Am J Physiol (1999) 276(3 Pt 2):R715–23. 10070131 10.1152/ajpregu.1999.276.3.R715

[105] Yaguchi K Kayaba S Soga H Yamagishi M Tamura A Kasahara S DNA fragmentation and detachment of enterocytes induced by anti-CD3 mAb-activated intraepithelial lymphocytes. Cell Tissue Res (2004) 315(1):71–84. 10.1007/s00441-003-0795-0 14579144

[106] Ogata M Itoh T Gamma/delta intraepithelial lymphocytes in the mouse small intestine. Anat Sci Int (2016) 91(4):301–12. 10.1007/s12565-016-0341-2 27056578

[107] Swamy M Abeler-Dorner L Chettle J Mahlakoiv T Goubau D Chakravarty P Intestinal intraepithelial lymphocyte activation promotes innate antiviral resistance. Nat Commun (2015) 6:7090. 10.1038/ncomms8090 25987506 PMC4479038

[108] Macpherson AJ Uhr T. Induction of protective IgA by intestinal dendritic cells carrying commensal bacteria. Science (2004) 303(5664):1662–5. 10.1126/science.1091334 15016999

[109] Tanaka Y Morita CT Tanaka Y Nieves E Brenner MB Bloom BR. Natural and synthetic non-peptide antigens recognized by human gamma delta T cells. Nature (1995) 375(6527):155–8. 10.1038/375155a0 7753173

[110] Medzhitov R Janeway CA Jr How does the immune system distinguish self from nonself? Semin Immunol (2000) 12(3):185–8; discussion 257–344. 10.1006/smim.2000.0230 10910738

[111] Sander LE Davis MJ Boekschoten MV Amsen D Dascher CC Ryffel B Detection of prokaryotic mRNA signifies microbial viability and promotes immunity. Nature (2011) 474(7351):385–9. 10.1038/nature10072 21602824 PMC3289942

[112] Slack E Balmer ML Fritz JH Hapfelmeier S. Functional flexibility of intestinal IgA – broadening the fine line. Front Immunol (2012) 3:100. 10.3389/fimmu.2012.00100 22563329 PMC3342566

[113] Palm NW de Zoete MR Cullen TW Barry NA Stefanowski J Hao L Immunoglobulin A coating identifies colitogenic bacteria in inflammatory bowel disease. Cell (2014) 158(5):1000–10. 10.1016/j.cell.2014.08.006 25171403 PMC4174347

[114] Fujihashi K McGhee JR Kweon MN Cooper MD Tonegawa S Takahashi I gamma/delta T cell-deficient mice have impaired mucosal immunoglobulin A responses. J Exp Med (1996) 183(4):1929–35. 10.1084/jem.183.4.1929 8666951 PMC2192480

[115] Wang L Kamath A Das H Li L Bukowski JF. Antibacterial effect of human V gamma 2V delta 2 T cells in vivo. J Clin Invest (2001) 108(9):1349–57. 10.1172/JCI13584 11696580 PMC209444

[116] Linterman MA Denton AE Divekar DP Zvetkova I Kane L Ferreira C CD28 expression is required after T cell priming for helper T cell responses and protective immunity to infection. Elife (2014) 3. 10.7554/eLife.03180 25347065 PMC4241536

[117] Lefrancois L. Phenotypic complexity of intraepithelial lymphocytes of the small intestine. J Immunol (1991) 147(6):1746–51. 1716278

[118] Van Houten N Mixter PF Wolfe J Budd RC. CD2 expression on murine intestinal intraepithelial lymphocytes is bimodal and defines proliferative capacity. Int Immunol (1993) 5(6):665–72. 10.1093/intimm/5.6.665 8102249

[119] Ohishi K Varnum-Finney B Bernstein ID. Delta-1 enhances marrow and thymus repopulating ability of human CD34(+)CD38(-) cord blood cells. J Clin Invest (2002) 110(8):1165–74. 10.1172/JCI16167 12393852 PMC150801

[120] Marriott CL Mackley EC Ferreira C Veldhoen M Yagita H Withers DR. OX40 controls effector CD4+ T-cell expansion, not follicular T helper cell generation in acute Listeria infection. Eur J Immunol (2014) 44(8):2437–47. 10.1002/eji.201344211 24771127 PMC4285916

[121] Wang HC Klein JR Multiple levels of activation of murine CD8(+) intraepithelial lymphocytes defined by OX40 (CD134) expression: effects on cell-mediated cytotoxicity, IFN-gamma, and IL-10 regulation. J Immunol (2001) 167(12):6717–23. 10.4049/jimmunol.167.12.6717 11739485

[122] Souza HS Elia CC Spencer J MacDonald TT. Expression of lymphocyte-endothelial receptor-ligand pairs, alpha4beta7/MAdCAM-1 and OX40/OX40 ligand in the colon and jejunum of patients with inflammatory bowel disease. Gut (1999) 45(6):856–63. 10.1136/gut.45.6.856 10562584 PMC1727744

[123] Stuber E Buschenfeld A Luttges J Von Freier A Arendt T Folsch UR. The expression of OX40 in immunologically mediated diseases of the gastrointestinal tract (celiac disease, Crohn’s disease, ulcerative colitis). Eur J Clin Invest (2000) 30(7):594–9. 10.1046/j.1365-2362.2000.00658.x 10886299

[124] Witherden DA Verdino P Rieder SE Garijo O Mills RE Teyton L The junctional adhesion molecule JAML is a costimulatory receptor for epithelial gammadelta T cell activation. Science (2010) 329(5996):1205–10. 10.1126/science.1192698 20813954 PMC2943937

[125] Verdino P Witherden DA Ferguson MS Corper AL Schiefner A Havran WL Molecular insights into gammadelta T cell costimulation by an anti-JAML antibody. Structure (2011) 19(1):80–9. 10.1016/j.str.2010.10.007 21220118 PMC3039130

[126] Weber DA Sumagin R McCall IC Leoni G Neumann PA Andargachew R Neutrophil-derived JAML inhibits repair of intestinal epithelial injury during acute inflammation. Mucosal Immunol (2014) 7(5):1221–32. 10.1038/mi.2014.12 24621992 PMC4340686

[127] Puddington L Olson S Lefrancois L. Interactions between stem cell factor and c-Kit are required for intestinal immune system homeostasis. Immunity (1994) 1(9):733–9. 10.1016/S1074-7613(94)80015-4 7534619

[128] Lodolce JP Boone DL Chai S Swain RE Dassopoulos T Trettin S IL-15 receptor maintains lymphoid homeostasis by supporting lymphocyte homing and proliferation. Immunity (1998) 9(5):669–76. 10.1016/S1074-7613(00)80664-0 9846488

[129] Kennedy MK Glaccum M Brown SN Butz EA Viney JL Embers M Reversible defects in natural killer and memory CD8 T cell lineages in interleukin 15-deficient mice. J Exp Med (2000) 191(5):771–80. 10.1084/jem.191.5.771 10704459 PMC2195858

[130] Schluns KS Nowak EC Cabrera-Hernandez A Puddington L Lefrancois L Aguila HL. Distinct cell types control lymphoid subset development by means of IL-15 and IL-15 receptor alpha expression. Proc Natl Acad Sci U S A (2004) 101(15):5616–21. 10.1073/pnas.0307442101 15060278 PMC397446

[131] Ma LJ Acero LF Zal T Schluns KS. Trans-presentation of IL-15 by intestinal epithelial cells drives development of CD8alphaalpha IELs. J Immunol (2009) 183(2):1044–54. 10.4049/jimmunol.0900420 19553528 PMC2706935

[132] Dubois S Mariner J Waldmann TA Tagaya Y. IL-15ralpha recycles and presents IL-15 in trans to neighboring cells. Immunity (2002) 17(5):537–47. 10.1016/S1074-7613(02)00429-6 12433361

[133] Malamut G El Machhour R Montcuquet N Martin-Lanneree S Dusanter-Fourt I Verkarre V IL-15 triggers an antiapoptotic pathway in human intraepithelial lymphocytes that is a potential new target in celiac disease-associated inflammation and lymphomagenesis. J Clin Invest (2010) 120(6):2131–43. 10.1172/JCI41344 20440074 PMC2877946

[134] Mackay LK Wynne-Jones E Freestone D Pellicci DG Mielke LA Newman DM T-box transcription factors combine with the cytokines TGF-beta and IL-15 to control tissue-resident memory T cell fate. Immunity (2015) 43(6):1101–11. 10.1016/j.immuni.2015.11.008 26682984

[135] Meresse B Chen Z Ciszewski C Tretiakova M Bhagat G Krausz TN Coordinated induction by IL-15 of a TCR-independent NKG2D signaling pathway converts CTL into lymphokine-activated killer cells in celiac disease. Immunity (2004) 21(3):357–66. 10.1016/j.immuni.2004.06.020 15357947

[136] Itsumi M Yoshikai Y Yamada H. IL-15 is critical for the maintenance and innate functions of self-specific CD8(+) T cells. Eur J Immunol (2009) 39(7):1784–93. 10.1002/eji.200839106 19544306

[137] DePaolo RW Abadie V Tang F Fehlner-Peach H Hall JA Wang W Co-adjuvant effects of retinoic acid and IL-15 induce inflammatory immunity to dietary antigens. Nature (2011) 471(7337):220–4. 10.1038/nature09849 21307853 PMC3076739

[138] Jabri B Sollid LM. Tissue-mediated control of immunopathology in coeliac disease. Nat Rev Immunol (2009) 9(12):858–70. 10.1038/nri2670 19935805

[139] Strengell M Matikainen S Siren J Lehtonen A Foster D Julkunen I IL-21 in synergy with IL-15 or IL-18 enhances IFN-gamma production in human NK and T cells. J Immunol (2003) 170(11):5464–9. 10.4049/jimmunol.170.11.5464 12759422

[140] Sarra M Cupi ML Monteleone I Franze E Ronchetti G Di Sabatino A IL-15 positively regulates IL-21 production in celiac disease mucosa. Mucosal Immunol (2013) 6(2):244–55. 10.1038/mi.2012.65 22785229

[141] Jameson SC Maintaining the norm: T-cell homeostasis. Nat Rev Immunol (2002) 2(8):547–56. 10.1038/nri853 12154374

[142] Laky K Lefrancois L Lingenheld EG Ishikawa H Lewis JM Olson S Enterocyte expression of interleukin 7 induces development of gammadelta T cells and Peyer’s patches. J Exp Med (2000) 191(9):1569–80. 10.1084/jem.191.9.1569 10790431 PMC2213426

[143] Hara T Shitara S Imai K Miyachi H Kitano S Yao H Identification of IL-7-producing cells in primary and secondary lymphoid organs using IL-7-GFP knock-in mice. J Immunol (2012) 189(4):1577–84. 10.4049/jimmunol.1200586 22786774

[144] Shalapour S Deiser K Kuhl AA Glauben R Krug SM Fischer A Interleukin-7 links T lymphocyte and intestinal epithelial cell homeostasis. PLoS One (2012) 7(2):e31939. 10.1371/journal.pone.0031939 22384106 PMC3288069

[145] Moore TA von Freeden-Jeffry U Murray R Zlotnik A. Inhibition of gamma delta T cell development and early thymocyte maturation in IL-7-/- mice. J Immunol (1996) 157(6):2366–73. 8805634

[146] Colpitts SL Stoklasek TA Plumlee CR Obar JJ Guo C Lefrancois L Cutting edge: the role of IFN-alpha receptor and MyD88 signaling in induction of IL-15 expression in vivo. J Immunol (2012) 188(6):2483–7. 10.4049/jimmunol.1103609 22327071 PMC3294000

[147] Yang H Spencer AU Teitelbaum DH. Interleukin-7 administration alters intestinal intraepithelial lymphocyte phenotype and function in vivo. Cytokine (2005) 31(6):419–28. 10.1016/j.cyto.2005.06.014 16102972

[148] Watanabe M Ueno Y Yajima T Okamoto S Hayashi T Yamazaki M Interleukin 7 transgenic mice develop chronic colitis with decreased interleukin 7 protein accumulation in the colonic mucosa. J Exp Med (1998) 187(3):389–402. 10.1084/jem.187.3.389 9449719 PMC2212121

[149] Taylor BC Zaph C Troy AE Du Y Guild KJ Comeau MR TSLP regulates intestinal immunity and inflammation in mouse models of helminth infection and colitis. J Exp Med (2009) 206(3):655–67. 10.1084/jem.20081499 19273626 PMC2699121

[150] Rochman Y Leonard WJ. The role of thymic stromal lymphopoietin in CD8+ T cell homeostasis. J Immunol (2008) 181(11):7699–705. 10.4049/jimmunol.181.11.7699 19017958 PMC2735224

[151] Rimoldi M Chieppa M Salucci V Avogadri F Sonzogni A Sampietro GM Intestinal immune homeostasis is regulated by the crosstalk between epithelial cells and dendritic cells. Nat Immunol (2005) 6(5):507–14. 10.1038/ni1192 15821737

[152] Watanabe T Kitani A Murray PJ Wakatsuki Y Fuss IJ Strober W. Nucleotide binding oligomerization domain 2 deficiency leads to dysregulated TLR2 signaling and induction of antigen-specific colitis. Immunity (2006) 25(3):473–85. 10.1016/j.immuni.2006.06.018 16949315

[153] Klimpel GR Langley KE Wypych J Abrams JS Chopra AK Niesel DW. A role for stem cell factor (SCF): c-Kit interaction(s) in the intestinal tract response to *Salmonella typhimurium* infection. J Exp Med (1996) 184(1):271–6. 10.1084/jem.184.1.271 8691142 PMC2192692

[154] Wang T Langley KE Gourley WK Klimpel GR. Stem cell factor (SCF) can regulate the activation and expansion of murine intraepithelial lymphocytes. Cytokine (2000) 12(3):272–80. 10.1006/cyto.1999.0551 10704255

[155] Hayday A Tigelaar R. Immunoregulation in the tissues by gammadelta T cells. Nat Rev Immunol (2003) 3(3):233–42. 10.1038/nri1030 12658271

[156] Ismail AS Severson KM Vaishnava S Behrendt CL Yu X Benjamin JL Gammadelta intraepithelial lymphocytes are essential mediators of host-microbial homeostasis at the intestinal mucosal surface. Proc Natl Acad Sci U S A (2011) 108(21):8743–8. 10.1073/pnas.1019574108 21555560 PMC3102410

[157] Roberts SJ Smith AL West AB Wen L Findly RC Owen MJ T-cell alpha beta + and gamma delta + deficient mice display abnormal but distinct phenotypes toward a natural, widespread infection of the intestinal epithelium. Proc Natl Acad Sci U S A (1996) 93(21):11774–9. 10.1073/pnas.93.21.11774 8876213 PMC38134

[158] Inagaki-Ohara K Dewi FN Hisaeda H Smith AL Jimi F Miyahira M Intestinal intraepithelial lymphocytes sustain the epithelial barrier function against *Eimeria vermiformis* infection. Infect Immun (2006) 74(9):5292–301. 10.1128/IAI.02024-05 16926423 PMC1594832

[159] Dalton JE Cruickshank SM Egan CE Mears R Newton DJ Andrew EM Intraepithelial gammadelta+ lymphocytes maintain the integrity of intestinal epithelial tight junctions in response to infection. Gastroenterology (2006) 131(3):818–29. 10.1053/j.gastro.2006.06.003 16952551

[160] Edelblum KL Singh G Odenwald MA Lingaraju A El Bissati K McLeod R Gammadelta intraepithelial lymphocyte migration limits transepithelial pathogen invasion and systemic disease in mice. Gastroenterology (2015) 148(7):1417–26. 10.1053/j.gastro.2015.02.053 25747597 PMC4685713

[161] Moretto M Durell B Schwartzman JD Khan IA Gamma delta T cell-deficient mice have a down-regulated CD8+ T cell immune response against *Encephalitozoon cuniculi* infection. J Immunol (2001) 166(12):7389–97. 10.4049/jimmunol.166.12.7389 11390490

[162] Chien YH Meyer C Bonneville M Gammadelta T cells: first line of defense and beyond. Annu Rev Immunol (2014) 32:121–55. 10.1146/annurev-immunol-032713-120216 24387714

[163] Ganz T. Defensins: antimicrobial peptides of innate immunity. Nat Rev Immunol (2003) 3(9):710–20. 10.1038/nri1180 12949495

[164] Mankertz J Tavalali S Schmitz H Mankertz A Riecken EO Fromm M Expression from the human occludin promoter is affected by tumor necrosis factor alpha and interferon gamma. J Cell Sci (2000) 113(Pt 11):2085–90. 10806119 10.1242/jcs.113.11.2085

[165] Dharakul T Rott L Greenberg HB. Recovery from chronic rotavirus infection in mice with severe combined immunodeficiency: virus clearance mediated by adoptive transfer of immune CD8+ T lymphocytes. J Virol (1990) 64(9):4375–82. 1974652 10.1128/jvi.64.9.4375-4382.1990 PMC247905

[166] Sydora BC Jamieson BD Ahmed R Kronenberg M. Intestinal intraepithelial lymphocytes respond to systemic lymphocytic choriomeningitis virus infection. Cell Immunol (1996) 167(2):161–9. 10.1006/cimm.1996.0023 8603424

[167] Muller S Buhler-Jungo M Mueller C. Intestinal intraepithelial lymphocytes exert potent protective cytotoxic activity during an acute virus infection. J Immunol (2000) 164(4):1986–94. 10.4049/jimmunol.164.4.1986 10657649

[168] Karst SM Wobus CE Lay M Davidson J Virgin HW IV. STAT1-dependent innate immunity to a Norwalk-like virus. Science (2003) 299(5612):1575–8. 10.1126/science.1077905 12624267

[169] Nice TJ Baldridge MT McCune BT Norman JM Lazear HM Artyomov M Interferon-lambda cures persistent murine norovirus infection in the absence of adaptive immunity. Science (2015) 347(6219):269–73. 10.1126/science.1258100 25431489 PMC4398891

[170] Hernandez PP Mahlakoiv T Yang I Schwierzeck V Nguyen N Guendel F Interferon-lambda and interleukin 22 act synergistically for the induction of interferon-stimulated genes and control of rotavirus infection. Nat Immunol (2015) 16(7):698–707. 10.1038/ni.3180 26006013 PMC4589158

[171] Mahlakoiv T Hernandez P Gronke K Diefenbach A Staeheli P Leukocyte-derived IFN-alpha/beta and epithelial IFN-lambda constitute a compartmentalized mucosal defense system that restricts enteric virus infections. PLoS Pathog (2015) 11(4):e1004782 10.1371/journal.ppat.1004782 25849543 PMC4388470

[172] Kuss SK Best GT Etheredge CA Pruijssers AJ Frierson JM Hooper LV Intestinal microbiota promote enteric virus replication and systemic pathogenesis. Science (2011) 334(6053):249–52. 10.1126/science.1211057 21998395 PMC3222156

[173] von Hertzen L Beutler B Bienenstock J Blaser M Cani PD Eriksson J Helsinki alert of biodiversity and health. Ann Med (2015) 47(3):218–25. 10.3109/07853890.2015.1010226 25904094

[174] Attia S Versloot CJ Voskuijl W van Vliet SJ Di Giovanni V Zhang L Mortality in children with complicated severe acute malnutrition is related to intestinal and systemic inflammation: an observational cohort study. Am J Clin Nutr (2016) 104(5):1441–9. 10.3945/ajcn.116.130518 27655441 PMC5081715

[175] Koga T McGhee JR Kato H Kato R Kiyono H Fujihashi K. Evidence for early aging in the mucosal immune system. J Immunol (2000) 165(9):5352–9. 10.4049/jimmunol.165.9.5352 11046071

[176] Glocker E Grimbacher B. Inflammatory bowel disease: is it a primary immunodeficiency? Cell Mol Life Sci (2012) 69(1):41–8. 10.1007/s00018-011-0837-9 21997382 PMC11114923

[177] Vinh DC Behr MA. Crohn’s as an immune deficiency: from apparent paradox to evolving paradigm. Expert Rev Clin Immunol (2013) 9(1):17–30. 10.1586/eci.12.87 23256761

[178] de Souza HS Fiocchi C. Immunopathogenesis of IBD: current state of the art. Nat Rev Gastroenterol Hepatol (2016) 13(1):13–27. 10.1038/nrgastro.2015.186 26627550

[179] Ryan P Kelly RG Lee G Collins JK O’Sullivan GC O’Connell J Bacterial DNA within granulomas of patients with Crohn’s disease – detection by laser capture microdissection and PCR. Am J Gastroenterol (2004) 99(8):1539–43. 10.1111/j.1572-0241.2004.40103.x 15307874

[180] Veldhoen M. Interleukin 17 is a chief orchestrator of immunity. Nat Immunol (2017) 18(6):612–21. 10.1038/ni.3742 28518156

[181] MacDonald TT Spencer J. Evidence that activated mucosal T cells play a role in the pathogenesis of enteropathy in human small intestine. J Exp Med (1988) 167(4):1341–9. 10.1084/jem.167.4.1341 2965735 PMC2188906

[182] Peluso I Pallone F Monteleone G. Interleukin-12 and Th1 immune response in Crohn’s disease: pathogenetic relevance and therapeutic implication. World J Gastroenterol (2006) 12(35):5606–10. 10.3748/wjg.v12.i35.5606 17007011 PMC4088159

[183] Watanabe M Ueno Y Yajima T Iwao Y Tsuchiya M Ishikawa H Interleukin 7 is produced by human intestinal epithelial cells and regulates the proliferation of intestinal mucosal lymphocytes. J Clin Invest (1995) 95(6):2945–53. 10.1172/JCI118002 7769137 PMC295983

[184] Liu Z Geboes K Colpaert S D’Haens GR Rutgeerts P Ceuppens JL. IL-15 is highly expressed in inflammatory bowel disease and regulates local T cell-dependent cytokine production. J Immunol (2000) 164(7):3608–15. 10.4049/jimmunol.164.7.3608 10725717

[185] McVay LD Li B Biancaniello R Creighton MA Bachwich D Lichtenstein G Changes in human mucosal gamma delta T cell repertoire and function associated with the disease process in inflammatory bowel disease. Mol Med (1997) 3(3):183–203. 9100225 PMC2230043

[186] Inagaki-Ohara K Chinen T Matsuzaki G Sasaki A Sakamoto Y Hiromatsu K Mucosal T cells bearing TCRgammadelta play a protective role in intestinal inflammation. J Immunol (2004) 173(2):1390–8. 10.4049/jimmunol.173.2.1390 15240735

[187] Kohyama M Nanno M Kawaguchi-Miyashita M Shimada S Watanabe M Hibi T Cytolytic and IFN-gamma-producing activities of gamma delta T cells in the mouse intestinal epithelium are T cell receptor-beta-chain dependent. Proc Natl Acad Sci U S A (1999) 96(13):7451–5. 10.1073/pnas.96.13.7451 10377435 PMC22106

[188] Kawaguchi-Miyashita M Shimada S Kurosu H Kato-Nagaoka N Matsuoka Y Ohwaki M An accessory role of TCRgammadelta (+) cells in the exacerbation of inflammatory bowel disease in TCRalpha mutant mice. Eur J Immunol (2001) 31(4):980–8. 10.1002/1521-4141(200104)31:4<980::AID-IMMU980>3.0.CO;2-U 11298322

[189] Kadivar M Petersson J Svensson L Marsal J CD8alphabeta+ gammadelta T Cells: a novel T cell subset with a potential role in inflammatory bowel disease. J Immunol (2016) 197(12):4584–92. 10.4049/jimmunol.1601146 27849165

[190] Hugot JP Chamaillard M Zouali H Lesage S Cezard JP Belaiche J Association of NOD2 leucine-rich repeat variants with susceptibility to Crohn’s disease. Nature (2001) 411(6837):599–603. 10.1038/35079107 11385576

[191] Ogura Y Bonen DK Inohara N Nicolae DL Chen FF Ramos R A frameshift mutation in NOD2 associated with susceptibility to Crohn’s disease. Nature (2001) 411(6837):603–6. 10.1038/35079114 11385577

[192] Girardin SE Hugot JP Sansonetti PJ. Lessons from Nod2 studies: towards a link between Crohn’s disease and bacterial sensing. Trends Immunol (2003) 24(12):652–8. 10.1016/j.it.2003.10.007 14644139

[193] Liu JZ van Sommeren S Huang H Ng SC Alberts R Takahashi A Association analyses identify 38 susceptibility loci for inflammatory bowel disease and highlight shared genetic risk across populations. Nat Genet (2015) 47(9):979–86. 10.1038/ng.3359 26192919 PMC4881818

[194] Watanabe T Kitani A Murray PJ Strober W. NOD2 is a negative regulator of toll-like receptor 2-mediated T helper type 1 responses. Nat Immunol (2004) 5(8):800–8. 10.1038/ni1092 15220916

[195] Fukushima K Masuda T Ohtani H Sasaki I Funayama Y Matsuno S Immunohistochemical characterization, distribution, and ultrastructure of lymphocytes bearing T-cell receptor gamma/delta in inflammatory bowel disease. Gastroenterology (1991) 101(3):670–8. 10.1016/0016-5085(91)90524-O 1860632

[196] Bucht A Soderstrom K Esin S Grunewald J Hagelberg S Magnusson I Analysis of gamma delta V region usage in normal and diseased human intestinal biopsies and peripheral blood by polymerase chain reaction (PCR) and flow cytometry. Clin Exp Immunol (1995) 99(1):57–64. 10.1111/j.1365-2249.1995.tb03472.x 7813110 PMC1534135

[197] McCarthy NE Bashir Z Vossenkamper A Hedin CR Giles EM Bhattacharjee S Proinflammatory Vdelta2+ T cells populate the human intestinal mucosa and enhance IFN-gamma production by colonic alphabeta T cells. J Immunol (2013) 191(5):2752–63. 10.4049/jimmunol.1202959 23904167

[198] Kuhl AA Pawlowski NN Grollich K Loddenkemper C Zeitz M Hoffmann JC. Aggravation of intestinal inflammation by depletion/deficiency of gammadelta T cells in different types of IBD animal models. J Leukoc Biol (2007) 81(1):168–75. 10.1189/jlb.1105696 17041003

[199] Andreu-Ballester JC Garcia-Ballesteros C Amigo V Ballester F Gil-Borras R Catalan-Serra I Microsporidia and its relation to Crohn’s disease. A retrospective study. PLoS One (2013) 8(4):e62107. 10.1371/journal.pone.0062107 23637975 PMC3630148

